# One‐year conditional survival of dogs and cats with invasive mammary carcinomas: A concept inspired from human breast cancer

**DOI:** 10.1111/vco.12655

**Published:** 2020-09-28

**Authors:** Florian Chocteau, Valentin Mordelet, Elie Dagher, Delphine Loussouarn, Jérôme Abadie, Frédérique Nguyen

**Affiliations:** ^1^ AMaROC (Animal Cancers, Models for Research in Comparative Oncology) Oniris, Nantes Atlantic College of Veterinary Medicine, Food Science and Engineering Nantes France; ^2^ Université de Nantes, Inserm, CRCINA Nantes France; ^3^ Department of Pathology University Hospital Nantes France; ^4^ Integrated Center for Oncology Nantes/Angers Saint‐Herblain France

**Keywords:** cat, conditional survival, dog, invasive mammary carcinoma, prognosis

## Abstract

Numerous studies have described the prognostic factors of canine and feline mammary carcinomas (MCs), that is, variables that predict patient survival after diagnosis. But how does survival estimation evolve in patients that escaped early death from their cancer? In human oncology, conditional survival (CS), the probability of surviving X further years when cancer patients have already survived Y years, is used to analyse cancer outcomes in a long‐term perspective. In this cohort of 344 dogs and 342 cats with surgically removed stage I to III invasive MCs, with a minimal follow‐up of 2 years, we calculated the 1‐year CS, that is, the probability for patients that have survived 1 year, to survive or to die from cancer during the subsequent year. The 1‐year conditional specific survival probabilities were 59% and 48% at diagnosis of invasive MC respectively in dogs and cats, and 80% and 52% in 1‐year surviving dogs and cats respectively, suggesting that 1‐year surviving dogs were relatively protected from cancer‐related death, whereas feline MCs remained life‐threatening cancers for longer periods of time. Among the most significant parameters associated with CS in surviving dogs and cats were the nodal stage and lymphovascular invasion, as well as patient age, cancer stage and margin status in surviving dogs. By comparison, tumour size and the histological grade did not significantly alter CS probabilities in surviving dogs and cats. Conditional survival may be considered a very interesting tool for veterinary practitioners to estimate the likely outcome of cancer survivors.

## 1 INTRODUCTION

Mammary carcinomas (MCs) are among the most common malignant tumours in female dogs and cats, with an annual incidence of 192 MCs for 100 000 dogs^1^ and 230 mammary tumours for 100 000 cats, of which 80% to 90% are malignant.^2^, ^3^ MCs and, especially, invasive MCs show aggressive behaviour with high metastatic propensity^4^, ^5^, ^6^, ^7^ and short survival times after surgical removal (median overall survival of 11 months for dogs^8^ and 8 to 12 months for cats with MCs).^7^, ^9^, ^10^, ^11^ However, these data are general, and MCs in both dogs and cats show heterogeneity according to numerous factors. Among these, the histological subtype,^8^, ^12^, ^13^, ^14^ the histological grade,^8^, ^9^, ^12^, ^15^, ^16^, ^17^, ^18^, ^19^, ^20^ the histological stage,^21^, ^22^ surgical margin status,^13^, ^14^, ^23^ and immunophenotype^24^, ^25^ are robust factors significantly associated with survival probabilities or disease‐free interval after diagnosis of invasive MCs. In human oncology and particularly in breast cancer, all of these factors also provide prognostic information,^26^, ^27^, ^28^, ^29^, ^30^, ^31^, ^32^ given to patients at time of diagnosis using median survival times or 5‐ and 10‐year survival probabilities.

In human oncology however, survival probabilities are better than in veterinary medicine because of earlier diagnosis and more efficient therapies, and follow‐up is more rigorous.^33^ In this context, medical oncologists use the tool of conditional survival (CS) that corresponds to the probability of surviving X further months, given that a patient has already survived Y months after cancer diagnosis.^34^ For example, in patients with pancreatic cancer, at diagnosis, the probability of surviving 5 years is 7%, but for patients who survive to the first year, this probability increases to 27%, and reaches 63% if they survive 3 years.^33^ CS can be divided into absolute CS and relative CS. Absolute CS is calculated from a single cohort of patients, whereas relative CS compares survival probabilities between a cohort and an age‐matched healthy reference population.

To our knowledge, very few studies have described CS in veterinary medicine: Bonnett et al described the probability for dogs of living at least 5, 7, 8 or 10 years according to their breed,^35^ as well as the probability for an 8‐year‐old dog of a given breed to be alive by age 10, data that can be considered as “conditional survival” although these dogs were not affected by a given disease entity. Kass et al described the survival probability of dogs and cats who had survived a resuscitation attempt for cardiac arrest.^36^ However, no studies have investigated CS in veterinary oncology. The aims of this study were (a) to describe the 1‐year absolute overall and cancer‐specific CS of female dogs and cats with invasive MCs, and (b) to analyse the influence of epidemiological, clinical, histological and immunohistochemical parameters on this CS.

## 2 METHODS

### 2.1 Patients

This retrospective study included 344 canine and 342 feline invasive MCs that were diagnosed between 2004 and 2010 and have been previously described.^8^, ^15^, ^24^ The owners' written consent and approval from the local animal welfare committee of Oniris were obtained prior to inclusion. Inclusion criteria comprised female dogs and cats, diagnosed with an invasive MC, surgically removed, with a minimal follow‐up of 2 years. Patients were excluded if records were incomplete, if distant metastases or any other malignant tumours were present at diagnosis (based on clinical examination, and medical imaging when available), or if adjuvant treatments were administered before or after surgery. Age, breed, reproductive and medical history, and outcomes were obtained through written questionnaires or telephone interviews with referring veterinarians and owners.

### 2.2 Follow‐up and CS definition

The outcome data were overall survival (OS, time from mastectomy to death from any cause), and cancer‐specific survival (SS, time from mastectomy to death attributable to the MC). Because the follow‐up duration was 2 years in this study, we restricted our analyses to dogs and cats that had survived for 1 year, in order to calculate their probability of being alive, dead, or dying from cancer in the subsequent year. Thus, in this study, the 1‐year CS is defined as the probability of surviving one further year depending on the number of months (0‐12) already survived after diagnosis. More specifically, we separately calculated the 1‐year conditional overall survival (COS) and the 1‐year conditional cancer‐specific survival (CSS). Either natural or by euthanasia, death was considered “cancer‐related” in this study if it followed locoregional recurrence, distant metastasis (proven by medical imaging or necropsy, or highly suspected on clinical examination), or cancer cachexia, in the absence of any identified intercurrent disease.

### 2.3 Histopathology

Formalin‐fixed paraffin‐embedded tumour samples were cut into 3 μm‐thick sections and stained with haematoxylin‐eosin‐saffron (HES). Compared with the more widely used HE stain, the use of saffron in HES stains collagen in orange, but otherwise does not modify the histologic interpretation. In case of multiple/multicentric invasive MCs, the carcinoma with the largest diameter upon histological section was selected for analysis; if tumour size was identical between two MCs in a given patient, the MC with the highest histological grade was considered. Histological subtypes were described according to the adapted World Health Organization classification system.^16^, ^23^ In cases that demonstrated more than one histological architecture, the less differentiated was chosen. Histological stages were defined as previously described^21^, ^22^ according to the pathological tumour size (pT with pT1 ≤ 20 mm and pT2 > 20 mm), pathological nodal stage (pN with pN1, presence of nodal metastasis; pN0, absence of nodal metastasis, and pNX, lymph node not removed), and lymphovascular invasion (LVI, with LVI+ indicating presence, and LVI− indicating absence of lymphovascular invasion). Other recorded data included the Elston and Ellis histological grade, local invasion of dermis or muscle, margin status, tumour‐associated inflammation, central necrosis, ulceration, and squamous differentiation, as previously described.^8^, ^15^

### 2.4 Immunohistochemistry

Immunohistochemistry (IHC) was performed using a Benchmark XT automated instrument (Ventana Medical Systems, Roche Diagnostics) as previously described,^8^, ^21^, ^22^, ^24^, ^37^ and further detailed in Table S1. The antibodies used were p63 (used as myoepithelial marker), pancytokeratin (used as marker of metastatic epithelial cells in lymph nodes), LMO2 (lymphendothelial marker to better assess tumour emboli), ER (Oestrogen Receptor α), PR (Progesterone Receptor), HER2 (Human Epidermal growth factor Receptor 2), and Ki‐67 (proliferation marker). ER and PR were considered positive at a threshold of ≥10% in both dogs and cats and Ki‐67 was considered high at a threshold of >33% for dogs and ≥42% for cats. Carcinomas were defined as luminal (ER ≥ 10% and/or PR ≥ 10%) or triple‐negative (ER < 10%, PR < 10%, HER2 scores 0‐2+).^38^

Four veterinary pathologists and one medical pathologist examined the HES and IHC slides blindly, and collective agreement was obtained about the histological types, grades, and immunophenotypes. Involvement of a MD pathologist was indeed of invaluable help for immunophenotype assessment, especially for HER2 scoring.

### 2.5 Statistical analyses

Statistical analyses were performed using the MedCalc (Ostend, Belgium) and Microsoft Excel (Redmond, United States) statistical software. Continuous variables were described as median, range, mean ± SD. Categorical variables were compared using the Pearson chi‐square test and continuous variables with an ANOVA test (one‐way analysis of variance). CS rates of two independent groups were compared using the Pearson chi‐square test with reporting of the odds ratio (OR), its confidence interval (95% CI) and the *P* value. A *P* value of strictly less than .05 was considered significant.

## 3 RESULTS

### 3.1 Characteristics of female dogs with MCs

Three hundred and forty‐four bitches fulfilled the inclusion criteria (Table S2), including 78 cross‐bred (23%), 49 Poodle (14%), 23 German Shepherd (7%), 19 Labrador Retriever (6%), 19 Brittany Spaniel (6%), 10 Yorkshire Terrier (3%), and 146 other pure breed (41%) dogs. The median overall survival time post‐diagnosis was 11.7 months (mean 17.2 ± 16.9 months, range 0‐86.9 months). Further details on follow‐up are available in the Supporting Information.

Because CS is calculated in surviving patients, it was important to examine what were the characteristics of survivors, compared with canine patients that died within 1 year post‐diagnosis (Table S2). One hundred and seventy‐seven (51%) dogs died in the first year post‐diagnosis, and 167 (49%) survived at least 1 year. Compared with surviving dogs, those that died during the first year of follow‐up were significantly older (*P* = .003), were more likely to harbour a multicentric MC (*P* = .0197), with a larger tumour size (*P* = .0002), with nodal metastasis (*P* < .0001) and/or lymphovascular invasion (*P* < .0001), and thus a more advanced histological stage at diagnosis (*P* < .0001). Their MC was more likely to be inflammatory or anaplastic (*P* = .0025), of higher histological grade (*P* < .0001), with more severe tumour‐associated inflammation (*P* < .0001), more common infiltrated surgical margins (*P* < .0001) and muscle infiltration (*P* = .0126), and a higher Ki‐67 proliferation index (*P* = .0039).

### 3.2 One‐year conditional overall survival of dogs with MCs

**FIGURE 1:**
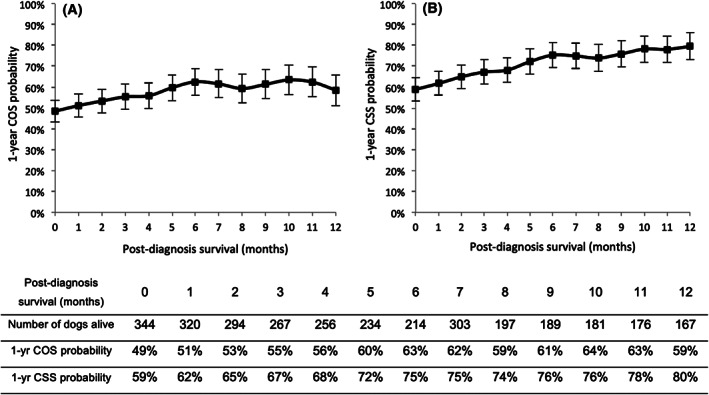
One‐year conditional survival of dogs according to the number of months survived after diagnosis of an invasive mammary carcinoma. A, 1‐year conditional overall survival (COS). The probability of being alive 1 year later slightly increased from diagnosis (49 ± 3%) to 12 months post‐diagnosis (59 ± 4%). B, 1‐year conditional cancer‐specific survival (CSS). The probability of dying from cancer during the following year was relatively high at diagnosis (41 ± 3%), but decreased with time, and was only 20 ± 3% in 12‐month survivors

In dogs, the probability of being alive 1 year after mastectomy was 49 ± 3% at diagnosis (Figure 1A), then the 1‐year conditional overall survival (COS) increased during the first 6 months after diagnosis, reaching 63 ± 3% in 6 month‐survivors, with a mean COS gain of 2.4% per month. From the 6th to the 12th month of survival, the COS probability levelled off (comprised between 59 ± 4% and 64 ± 4%).

We then analysed which parameters influenced conditional overall survival in surviving dogs. At diagnosis, 8 parameters were significantly associated with higher probabilities of being dead 1 year later (Table 1): an older age at diagnosis (OR = 1.90, *P* < .01), pT2 (OR = 2.22, *P* < .001), pN1 (OR = 2.97, *P* < .001), a histological stage III (OR = 6.48, *P* < .001), a histological grade III (OR = 2.31, *P* < .001), LVI+ (OR = 6.02, *P* < .001), positive margins (OR = 3.08, *P* < .001), and a high proliferation index (Ki‐67 > 33%, OR = 1.92, *P* < .01). With increasing numbers of months survived after diagnosis and up to 12 months after diagnosis, all of these factors, except pT (that lost its prognostic value after the 11th month of survival), were still significantly associated with COS probabilities (Figure S1). However, the odds ratios tended to decrease for the histological stage (OR = 2.35 for stage III in 12‐month survivors), LVI (OR = 2.32 for LVI+ in 12‐month survivors), and margin status (OR = 2.10 for positive margins in 12‐month survivors). Although the immunophenotype of canine MCs was not significantly prognostic at diagnosis, dogs with triple‐negative carcinomas had significantly lower COS after 7 months of survival than dogs with luminal MCs (OR = 2.85 in 9‐month survivors, OR = 2.20 in 12‐month survivors with triple‐negative MC, *P* < .05) (Table 1 and Figure S1H).

**TABLE 1:** Odds ratios of clinical‐pathologic parameters that influenced conditional overall survival of dogs according to duration of survival (in months) after diagnosis

Months after diagnosis		0	3	6	9	12
Age at diagnosis (years)	≤ 11.7 years, OR = 1.00	N = 227	N = 175	N = 146	N = 131	N = 122
	> 11.7 years, OR 95% CI	**1.90** ** 1.20‐3.00 N = 116	**3.12** *** 1.84‐5.28 N = 91	**3.05** *** 1.67‐5.55 N = 67	**3.24** *** 1.70‐6.18 N = 57	**2.74** ** 1.35‐5.57 N = 44
Pathologic tumour size (pT)	pT1, OR = 1.00	N = 160	N = 136	N = 119	N = 106	N = 94
	pT2, OR 95% CI	**2.22** *** 1.44‐3.42 N = 184	**2.24** ** 1.37‐3.66 N = 131	**1.99** * 1.13‐3.50 N = 95	**2.06** ** 1.14‐3.74 N = 83	1.59 0.85‐2.97 N = 73
Pathological nodal stage (pN)	pN0 or pNX, OR = 1.00	N = 270	N = 219	N = 185	N = 162	N = 146
	pN1, OR 95% CI	**2.97** *** 1.70‐5.20 N = 74	**3.04** *** 1.57‐5.88 N = 48	**2.22** * 1.01‐4.90 N = 29	**3.22** ** 1.38‐7.50 N = 27	**2.61** * 1.02‐6.28 N = 21
Histological stage	Stage I‐II, OR = 1.00	N = 160	N = 146	N = 133	N = 123	N = 115
	Stage III, OR 95% CI	**6.48** *** 4.05‐10.38 N = 184	**3.79** *** 2.28‐6.31 N = 121	**2.94** ** 1.65‐5.24 N = 81	**3.08** ** 1.66‐5.74 N = 66	**2.35** * 1.21‐4.59 N = 52
Histological grade	Grade I‐II, OR = 1.00	N = 123	N = 107	N = 90	N = 82	N = 76
	Grade III, OR 95% CI	**2.31** *** 1.47‐3.63 N = 221	**1.63** * 0.99‐2.69 N = 160	**2.26** ** 1.26‐4.07 N = 124	**2.04** * 1.11‐3.75 N = 107	**1.90** * 1.01‐3.58 N = 91
Lymphovascular invasion	LVI−, OR = 1.00	N = 177	N = 161	N = 141	N = 131	N = 122
	LVI+, OR 95% CI	**6.02** *** 3.77‐9.61 N = 167	**2.74** *** 1.65‐4.54 N = 106	**2.56** ** 1.43‐4.59 N = 73	**2.42** ** 1.29‐4.56 N = 58	**2.32** * 1.15‐4.65 N = 45
Margin status	Negative, OR = 1.00	N = 189	N = 164	N = 141	N = 128	N = 115
	Positive, OR 95% CI	**3.08** *** 1.98‐4.80 N = 155	**2.49** *** 1.50‐4.12 N = 103	**2.34** ** 1.31‐4.20 N = 73	**2.35** ** 1.26‐4.39 N = 61	**2.10** * 1.08‐4.08 N = 52
Ki‐67 index	Ki‐67 ≤ 33%, OR = 1.00	N = 161	N = 129	N = 111	N = 99	N = 92
	Ki‐67 > 33%, OR 95% CI	**1.92** ** 1.25‐2.94 N = 183	**2.46** *** 1.50‐4.04 N = 138	**2.78** *** 1.56‐4.92 N = 103	**3.06** *** 1.66‐5.63 N = 90	**2.47** ** 1.31‐4.65 N = 7
Immunophenotype	Luminal, OR = 1.00	N = 82	N = 75	N = 57	N = 50	N = 43
	Triple‐negative, OR 95% CI	1.23 0.75‐2.02 N = 262	0.89 0.52‐1.52 N = 192	1.58 0.82‐3.02 N = 157	**2.85** * 1.35‐6.03 N = 139	**2.20** * 1.04‐4.68 N = 124

*Note*: Odds ratios (OR) expressed with 95% confidence interval (95% CI).

^*^ *P* < .05.

^**^ *P* < .01.

^***^ *P* < .001.

### 3.3 One‐year conditional specific survival of dogs with MCs

The 1‐year conditional CSS increased from time of diagnosis (59 ± 3%) to 6 months after (75 ± 3%) with a mean CSS gain of 2.75% per month (Figure 1B). After the sixth month, the CSS tended to stabilize (between 74 ± 3% and 80 ± 3%).

At diagnosis, the same eight parameters associated with overall survival were significantly associated with CSS (Table 2): age at diagnosis (OR = 1.86, *P* < .01), pT (OR = 1.90, *P* < .001), pN (OR = 3.22, *P* < .001), the histological stage (OR = 7.29, *P* < .001), histological grade (OR = 2.26, *P* < .001), LVI (OR = 6.90, *P* < .001), margin status (OR = 2.55, *P* < .001), and the Ki‐67 proliferation index (OR = 2.67, *P* < .001). The odds ratios tended to decrease with survival time post‐diagnosis for the histological stage (OR = 3.53 for stage III in 12‐month survivors) and LVI (OR = 2.56 for LVI+ in 12‐month survivors). Some prognosticators at diagnosis lost their influence on CSS in surviving dogs: pT after the third month (Figure S2B), the histological grade after the first month, the Ki‐67 index after the 10th month (Figure S2G). Regarding the immunophenotype, although not significantly prognostic at diagnosis, a triple‐negative MC in dogs was significantly associated with lower CSS than luminal MCs after 7 months (OR = 3.43 in 9‐month survivors with triple‐negative MC, *P* < .01, Table 2 and Figure S2H).

**TABLE 2:** Odds ratios of clinical‐pathologic parameters that influenced conditional specific survival of dogs according to duration of survival (in months) after diagnosis

Months after diagnosis		0	3	6	9	12
Age at diagnosis (years)	≤ 11.7 years, OR = 1.00	N = 227	N = 175	N = 146	N = 131	N = 122
	> 11.7 years, OR 95% CI	**1.86** ** 1.18‐2.93 N = 116	**3.28** *** 1.92‐5.63 N = 91	**3.31** *** 1.73‐6.35 N = 67	**2.95** ** 1.46‐5.93 N = 57	**2.80** ** 1.26‐6.18 N = 44
Pathologic tumour size (pT)	pT1, OR = 1.00	N = 160	N = 136	N = 119	N = 106	N = 94
	pT2, OR 95% CI	**1.90** *** 1.22‐2.95 N = 184	**1.55** * 0.93‐2.59 N = 131	1.04 0.56‐1.94 N = 95	0.82 0.42‐1.63 N = 83	0.72 0.33‐1.59 N = 73
Pathological nodal stage (pN)	pN0 or pNX, OR = 1.00	N = 270	N = 219	N = 185	N = 162	N = 146
	pN1, OR 95% CI	**3.22** *** 1.88‐5.49 N = 74	**3.96** *** 2.07‐7.57 N = 48	**3.03** ** 1.35‐6.81 N = 29	**4.34** *** 1.86‐10.15 N = 27	**3.84** ** 1.47‐10.06 N = 21
Histological stage	Stage I‐II, OR = 1.00	N = 160	N = 146	N = 133	N = 123	N = 115
	Stage III, OR 95% CI	**7.29** *** 4.43‐12.00 N = 184	**5.19** *** 2.98‐9.05 N = 121	**4.83** *** 2.49‐9.39 N = 81	**4.10** *** 2.04‐8.24 N = 66	**3.53** *** 1.62‐7.67 N = 52
Histological grade	Grade I‐II, OR = 1.00	N = 123	N = 107	N = 90	N = 82	N = 76
	Grade III, OR 95% CI	**2.26** *** 1.41‐3.62 N = 221	1.29 0.76‐2.18 N = 160	1.46 0.77‐2.78 N = 124	1.27 0.64‐2.51 N = 107	0.95 0.45‐2.02 N = 91
Lymphovascular invasion	LVI−, OR = 1.00	N = 177	N = 161	N = 141	N = 131	N = 122
	LVI+, OR 95% CI	**6.90** *** 4.26‐11.17 N = 167	**3.59** *** 2.11‐6.13 N = 106	**3.84** *** 2.01‐7.35 N = 73	**2.78** ** 1.39‐5.56 N = 58	**2.56** * 1.15‐5.68 N = 45
Margin status	Negative, OR = 1.00	N = 189	N = 164	N = 141	N = 128	N = 115
	Positive, OR 95% CI	**2.55** *** 1.64‐3.96 N = 155	1.65 0.98‐2.77 N = 103	1.66 0.88‐3.13 N = 73	**1.97** * 0.99‐3.90 N = 61	**2.21** * 1.03‐4.72 N = 52
Ki‐67 index	Ki‐67 ≤ 33%, OR = 1.00	N = 161	N = 129	N = 111	N = 99	N = 92
	Ki‐67 > 33%, OR 95% CI	**2.67** *** 1.71‐4.18 N = 183	**3.22** *** 1.86‐5.56 N = 138	**3.70** *** 1.88‐7.28 N = 103	**3.16** ** 1.55‐6.43 N = 90	2.14 0.98‐4.66 N = 75
Immunophenotype	Luminal, OR = 1.00	N = 82	N = 75	N = 57	N = 50	N = 43
	Triple‐negative, OR 95% CI	1.37 0.82‐2.29 N = 262	1.17 0.66‐2.08 N = 192	1.92 0.88‐4.21 N = 157	**3.43** ** 1.29‐9.11 N = 139	**3.97** * 1.21‐13.10 N = 124

*Note*: Odds ratios (OR) expressed with 95% confidence interval (95% CI).

^*^ *P* < .05.

^**^ *P* < .01.

^***^ *P* < .001.

### 3.4 Characteristics of female cats with MCs

Three hundred and forty‐two queens fulfilled the inclusion criteria (Table S3), with 297 Domestic Shorthair or Longhair cats (87%), 17 Siamese (5%), 6 Persian (2%) and 22 other pure‐breed or cross‐bred cats (6%). The median overall survival time post‐diagnosis was 11.8 months (mean 15.5 ± 13.4 months, range 0‐73.2 months). Detailed follow‐up is available as Supporting Information.

One hundred and seventy‐five (51%) cats died in the first year post‐diagnosis. At initial presentation, compared with queens that survived at least 1 year, cats that died within the first year of follow‐up had a MC of larger pathologic tumour size (*P* < .001), more likely to be pN+ (*P* < .0001) and/or LVI+ (*P* < .0001), and thus of more advanced histological stage (*P* < .0001), and also more likely to be grade III (*P* = .0168), with dermal infiltration (*P* < .0001), cutaneous ulceration (*P* = .0025), and/or positive margins (*P* = .0309) (Table S3).

### 3.5 One‐year conditional overall survival of cats with MCs

**FIGURE 2:**
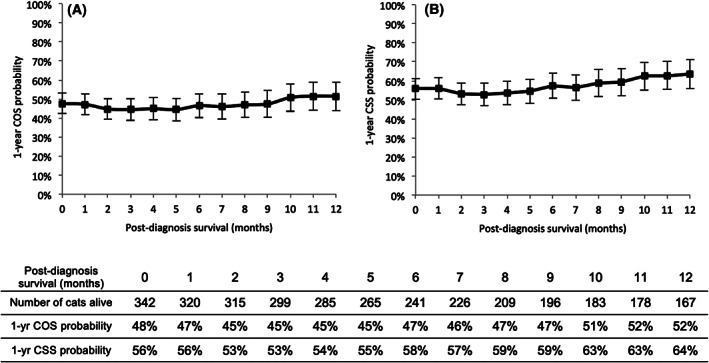
One‐year conditional survival of cats according to the number of months survived after diagnosis of an invasive mammary carcinoma. A, 1‐year conditional overall survival (COS). The probability of being alive 1 year later was stable from the time of diagnosis (t0, 48 ± 3%) to t0 + 12 months (52 ± 4%). B, 1‐year conditional cancer‐specific survival (CSS). Twelve‐month surviving cats had almost the same cancer‐specific survival probability (64 ± 4%) as at diagnosis (56 ± 3%)

The 1‐year conditional overall survival probabilities were stable from diagnosis (48 ± 3%) to 12 months of survival post‐diagnosis (52 ± 4%) (Figure 2A).

At diagnosis, seven parameters were significantly associated with higher probabilities for feline patients of being dead 1 year later: pT2 (OR = 3.35, *P* < .001), pN1 (OR = 2.34, *P* < .001), a histological stage III (OR = 2.72, *P* < .001), a histological grade III (OR = 1.87, *P* < .01), LVI+ (OR = 3.20, *P* < .001), positive margins (OR = 1.80, *P* < .01), and immunophenotype (OR = 4.19 for PR− ER+ and OR = 3.26 for PR− ER− MCs compared with PR+ MCs, *P* < .05) (Table 3 and Figure S3). However, with months of post‐diagnosis survival, these parameters lost their influence on COS: as early as the first month of survival for the immunophenotype (Figure S3G), after month 2 for the histological grade (Figure S3D), after month 6 for pT, the histological stage and LVI (Figure S3A,C,E) and after month 7 for surgical margins (Figure S3F). Only pN, the histological stage and LVI remained significant predictors of death within the subsequent year in 12‐month surviving cats (OR = 2.30 for pN1, OR = 1.91 for stage III, OR = 1.96 for LVI+, *P* < .05, Table 3 and Figure S3B,C,E).

**TABLE 3:** Clinical‐pathologic parameters that influenced conditional overall survival of cats according to duration of survival (in months) after diagnosis

Months after diagnosis		0	3	6	9	12
Pathologic tumour size (pT)	pT1, OR = 1.00	N = 188	N = 170	N = 153	N = 129	N = 115
	pT2, OR 95% CI	**3.35** *** 2.13‐5.25 N = 151	**2.79** *** 1.72‐4.54 N = 126	**1.76** * 1.02‐3.05 N = 85	1.49 0.81‐2.73 N = 64	1.05 0.54‐2.05 N = 49
Pathological nodal stage (pN)	pN0 or pNX, OR = 1.00	N = 245	N = 215	N = 183	N = 155	N = 133
	pN1, OR 95% CI	**2.34** *** 1.43‐3.82 N = 97	**2.57** *** 1.49‐4.42 N = 84	**2.07** * 1.12‐3.85 N = 58	2.01 0.98‐4.13 N = 41	**2.30** * 1.05‐5.02 N = 34
Histological stage	Stage I‐II, OR = 1.00	N = 161	N = 149	N = 138	N = 119	N = 99
	Stage III, OR 95% CI	**2.72** *** 1.75‐4.21 N = 181	**2.30** *** 1.44‐3.67 N = 150	**1.65** * 0.99‐2.77 N = 103	1.48 0.83‐2.64 N = 77	**1.91** * 1.02‐3.56 N = 68
Histological grade	Grade I‐II, OR = 1.00	N = 182	N = 168	N = 136	N = 120	N = 102
	Grade III, OR 95% CI	**1.87** ** 1.22‐2.88 N = 160	1.58 0.99‐2.51 N = 131	1.29 0.78‐2.16 N = 105	0.92 0.52‐1.64 N = 76	0.95 0.51‐1.77 N = 65
Lymphovascular invasion	LVI−, OR = 1.00	N = 174	N = 162	N = 149	N = 129	N = 108
	LVI+, OR 95% CI	**3.20** *** 2.05‐4.98 N = 168	**2.43** *** 1.52‐3.90 N = 137	**1.75** * 1.03‐2.97 N = 92	1.55 0.85‐2.83 N = 67	**1.96** * 1.03‐3.74 N = 59
Margin status	Negative, OR = 1.00	N = 169	N = 151	N = 129	N = 108	N = 93
	Positive, OR 95% CI	**1.80** ** 1.17‐2.76 N = 173	**1.91** ** 1.20‐3.03 N = 148	**1.85** * 1.10‐3.09 N = 112	1.62 0.92‐2.85 N = 88	1.65 0.89‐3.05 N = 74
Immunophenotype	PR+ any ER, OR = 1.00	N = 16	N = 16	N = 16	N = 14	N = 12
	ER+ PR−, OR 95% CI	**4.19** * 1.25‐14.09 N = 84	2.18 0.71‐6.70 N = 67	1.69 0.55‐5.17 N = 58	1.97 0.57‐6.85 N = 44	1.48 0.40‐5.51 N = 37
	ER− PR−, OR 95% CI	**3.26** * 1.02‐10.40 N = 242	2.16 0.76‐6.17 N = 216	1.46 0.52‐4.12 N = 167	2.14 0.68‐6.71 N = 138	1.31 0.39‐4.35 N = 118

*Note*: Odds ratios (OR) expressed with 95% confidence interval (95% CI).

^*^ *P* < .05.

^**^ *P* < .01.

^***^ *P* < .001.

### 3.6 One‐year conditional specific survival of cats with MCs

The 1‐year conditional CSS probabilities were also stable from diagnosis (56 ± 3%) to 12 months later (64 ± 4%) (Figure 2B).

At diagnosis, the same seven parameters associated with overall survival were significantly associated with the risk of dying from cancer during the subsequent year (Table 4): a larger pT (OR = 3.39, *P* < .001), a positive pN (OR = 2.56, *P* < .001), a histological stage III (OR = 2.65, *P* < .001), a histological grade III (OR = 1.60, *P* < .01), the presence of LVI (OR = 3.31, *P* < .001), positive margins (OR = 1.82, *P* < .01), and a PR‐negative phenotype (OR = 5.93 for PR− ER+ and OR = 5.05 for PR− ER− compared with PR+ MCs, *P* < .05). However, as cats survived a few months post‐diagnosis, most of these parameters lost their significant influence on CSS: the histological grade after the second month (Figure S4D), the immunophenotype after month 4 (Figure S4G), pT and margin status after month 6 (Figure S4AF). Only pN, the histological stage and LVI remained significantly associated with CSS regardless of the number of months survived after diagnosis (OR = 2.72 for pN1, OR = 2.83 for stage III, and OR = 2.73 for LVI+ MCs in 12‐month survivors, *P* < .01) (Table 4 and Figure S4B,C,E).

**TABLE 4:** Clinical‐pathologic parameters that influenced conditional specific survival of cats according to duration of survival (in months) after diagnosis

Months after diagnosis		0	3	6	9	12
Pathologic tumour size (pT)	pT1, OR = 1.00	N = 188	N = 170	N = 153	N = 129	N = 115
	pT2, OR 95% CI	**3.39** *** 2.16‐5.31 N = 151	**3.27** *** 2.02‐5.29 N = 126	**2.23** * 1.30‐3.82 N = 85	1.26 0.69‐2.32 N = 64	0.72 0.35‐1.46 N = 49
Pathological nodal stage (pN)	pN0 or pNX, OR = 1.00	N = 245	N = 215	N = 183	N = 155	N = 133
	pN1, OR 95% CI	**2.56** *** 1.58‐4.15 N = 97	**2.85** *** 1.68‐4.82 N = 84	**2.43** ** 1.33‐4.44 N = 58	**3.04** ** 1.49‐6.20 N = 41	**2.72** ** 1.26‐5.86 N = 34
Histological stage	Stage I‐II, OR = 1.00	N = 161	N = 149	N = 138	N = 119	N = 99
	Stage III, OR 95% CI	**2.65** *** 1.70‐4.13 N = 181	**2.49** *** 1.56‐3.97 N = 150	**1.90** * 1.13‐3.20 N = 103	**2.30** ** 1.27‐4.14 N = 77	**2.83** ** 1.47‐5.44 N = 68
Histological grade	Grade I‐II, OR = 1.00	N = 182	N = 168	N = 136	N = 120	N = 102
	Grade III, OR 95% CI	**1.60** * 1.04‐2.46 N = 160	1.52 0.96‐2.41 N = 131	1.30 0.78‐2.18 N = 105	0.99 0.55‐1.78 N = 76	0.98 0.51‐1.88 N = 65
Lymphovascular invasion	LVI−, OR = 1.00	N = 174	N = 162	N = 149	N = 129	N = 108
	LVI+, OR 95% CI	**3.31** *** 2.12‐5.17 N = 168	**2.78** *** 1.74‐4.45 N = 137	**2.12** ** 1.25‐3.59 N = 92	**2.32** ** 1.27‐4.25 N = 67	**2.73** ** 1.41‐5.29 N = 59
Margin status	Negative, OR = 1.00	N = 169	N = 151	N = 129	N = 108	N = 93
	Positive, OR 95% CI	**1.82** ** 1.18‐2.80 N = 173	**2.02** ** 1.27‐3.20 N = 148	**1.82** * 1.09‐3.06 N = 112	1.36 0.77‐2.41 N = 88	1.20 0.64‐2.27 N = 74
Immunophenotype	PR+ any ER, OR = 1.00	N = 16	N = 16	N = 16	N = 14	N = 12
	ER+ PR−, OR 95% CI	**5.93** ** 1.34‐26.15 N = 84	**3.14** * 0.87‐11.30 N = 67	3.11 0.85‐11.37 N = 58	4.49 0.96‐21.03 N = 44	3.87 0.80‐18.68 N = 37
	ER− PR−, OR 95% CI	**5.05** * 1.19‐21.38 N = 242	**3.49** * 1.03‐11.82 N = 216	2.73 0.80‐9.33 N = 167	3.67 0.85‐15.87 N = 138	2.17 0.49‐9.58 N = 118

*Note*: Odds ratios (OR) expressed with 95% confidence interval (95% CI).

^*^ *P* < .05.

^**^ *P* < .01.

^***^ *P* < .001.

## 4 DISCUSSION

This study evaluated the 1‐year absolute CS, regarding both all‐cause and cancer‐specific death, of a large cohort of 344 female dogs and 342 female cats according to the number of months survived after a diagnosis of stage I‐III invasive MC. To our knowledge, this is the first description of the concept of CS in veterinary oncology. However, previous studies in other fields of veterinary medicine mentioned a concept closely related to CS: Kass et al studied the 3‐day CS of dogs and cats after a cardiac arrest, the “condition” being that they had survived initial cardiopulmonary resuscitation,^36^ and Bonnett et al reported on the 2‐year CS of 8‐year‐old dogs, diseased or not, according to their breed.^35^ In human oncology and particularly in women with breast cancer, very few studies have dealt with absolute CS: most of the authors analyse relative CS, that is, the mortality rates of patients who have survived cancer relative to the mortality rates of age‐matched individuals of a reference population.

In this study, the 1‐year COS of cats with MC did not significantly improve over time, whereas in dogs, the 1‐year COS mildly increased with months already survived. Indeed, 12‐month surviving dogs had a +10% gain in their probability of being alive 1 year later (59 ± 4%) compared with the time of diagnosis (49 ± 3%). The same trend is observed in human breast cancer, although absolute data are not comparable because of far higher survival probabilities in humans than in animals. The 5‐year relative CS of breast cancer patients only marginally increases with years of survival, with a gain comprised between +3% and +5% in 5‐year survivors.^33^, ^39^, ^40^, ^41^, ^42^ In that respect, breast cancer markedly differs from lung, pancreatic or gastric cancer, characterized by relative CS gains comprised between +57% and +82% in 5 year‐survivors.^33^, ^40^ In the cats of the present study, the absence of COS improvement over time reflected the fact that dying during the first year post‐diagnosis was approximately as likely (175/342 cats, 51%) as dying during the second year (87/167 cats, 52%). It is possible, but not demonstrable in the present study with 2‐year follow‐up, that CS of cats with MC increases after 2 years of survival, as it increased in dogs of the present study after 1 year of survival, and as it does in human cancer patients after 5 years of survival.

Data on conditional overall survival carry the bias of patient age, because with increasing numbers of months survived, the patient is growing older, and thus has a growing probability of dying, regardless of the cause. Thus, cancer‐specific CS that focuses on cancer‐related deaths only, is probably more relevant than COS. In cats with MCs, the 1‐year CSS marginally increased, with a +8% gain in 12 months (from 56 ± 3% at diagnosis to 64 ± 4% in 12‐month survivors), whereas in dogs, the probability of dying from cancer in the subsequent year decreased 2‐fold in 12‐month survivors (20 ± 3%) compared with dogs at diagnosis (41 ± 3%). Actually, in dogs, most cancer‐related deaths occurred during the first year of follow‐up (72%, 130/181 cancer‐related deaths), whereas in cats, cancer‐related deaths were more common than in dogs, but less concentrated during the first year post‐diagnosis (63%, 142/226 cancer‐related deaths occurred within the first year). However, canine and feline MCs of the present study did not significantly differ by patient age or cancer stage at diagnosis, so early mortality in dogs could not be explained by an older age or higher extent of their mammary cancers compared with cats. One can suppose that the more common late deaths observed in cats compared with dogs could be related to species differences. This might be because of a higher proportion of feline patients with dormant micrometastases, susceptible to cancer resurgence at any time post‐diagnosis, or to a longer dormancy of micrometastases in cats than in dogs. Alternatively, the 1‐year surviving dogs might be those with effective antitumour immune control, for genetic and/or environmental reasons, explaining their low probabilities of cancer‐related death after 1 year of survival.

Multiple clinical‐pathologic parameters affected CS in the present study. In dogs, an older age at diagnosis was significantly associated with lower CSS from diagnosis to 12 months later (OR = 1.86‐3.31, *P* < .01). On the contrary, both in cats of the present study and in women with breast cancer,^33^, ^42^, ^43^, ^44^, ^45^ patient age does not significantly influence CSS, except in the study by Janssen et al, in which better 5‐year CS in 5‐year survivors was achieved for women aged 45 to 64 years compared with younger and older women.^41^

In canine and feline MCs, tumour size was a very strong prognosticator associated with specific survival at diagnosis, as previously reported in prognostic studies.^14^, ^20^, ^46^ However, our results indicate that pT was no longer prognostic in terms of SS in surviving patients, from the third month post‐diagnosis in dogs and from the sixth month in cats. In other words, most cancer‐related deaths because of large MCs occurred early, and >6‐month survivors were dogs and cats that had escaped the early lethal effect of tumour size. The results were completely different with LVI and pN, which are both strong prognostic factors of canine and feline MCs at diagnosis,^13^, ^15^, ^19^, ^25^, ^47^, ^48^, ^49^, ^50^, ^51^, ^52^, ^53^, ^54^ but which were also strongly associated with CS in the present study, including in 12‐month survivors. As LVI and pN reflect the metastatic spread of cancer, one can hypothesize that even long‐term surviving cats and dogs remain at high risk of cancer‐related death because of distant metastases if their MC was LVI+ and/or pN+.

In dogs and cats, the histological stage of invasive MCs, derived from pT, pN and LVI,^21^, ^22^ was significantly associated with SS at diagnosis (we compared stage I‐II MCs: any pT, pN0‐pNX and LVI−, with stage III MCs: any pT, pN+ and/or LVI+). In both species, stage I to II MCs showed a relatively stable CSS probability over time (+5% gain in CSS in dogs and cats, from diagnosis to 12 months after). By comparison, stage III MCs were associated with a +27% gain in CSS in dogs from diagnosis to 12 months after, whereas in cats stage III MCs showed also a relatively stable CSS probability over time (+5% gain in CSS from diagnosis to 12 months after); in both species, cancer stage still impacted CSS in 12‐month survivors. These observations in dogs correlate with those reported in human breast cancer, in which lower stages do not significantly affect the relative CS, whereas women with higher‐stage breast cancers have a significant improvement of their relative CS if they have survived 5 years.^42^, ^43^, ^44^, ^45^, ^55^, ^56^ In other words, the more advanced stage at diagnosis, the more survival time means “good news” for the patient, that is, decreased probability of dying from cancer afterwards.

In our cohort as in human breast cancer,^45^ the histological grade was significantly associated with specific survival at diagnosis, but not in surviving patients. Grade I to II MCs showed a relatively stable CSS over time (+8% in dogs and +3% in cats within 12 months post‐diagnosis), whereas grade III MCs were associated with a +28% gain in CSS in dogs and +15% in cats during the first year of follow‐up. In women, the CSS improvement over time observed for high grade breast cancers could be related to more aggressive therapy and better response to treatment of grade III compared with grade I breast cancers.^45^ In the present cohort of animals that had surgery as a single therapy, this increase in CSS with survival time could be because of an earlier death of patients with higher grade MCs.

In dogs (but not cats) with MCs, the Ki‐67 proliferation index was significantly associated with CSS probabilities during the first 10 months post‐diagnosis (difference in CSS comprised between −15% and −25% for highly proliferative compared with slowly proliferative MCs). Afterwards, Ki‐67 was not significantly associated with CSS because there had been a +25% gain in CSS in 12 months for highly proliferative MCs. In women with breast cancer, the Ki‐67 proliferation index (at 14% cut‐off) is not significantly associated with relative CS of 5‐year surviving patients.^56^

With respect to immunophenotype, PR positivity of feline MCs was a protecting factor during the first 4 months of follow‐up, and at months 9 and 10, with higher CSS probabilities associated with PR+ MCs than ER+ PR− and ER− PR− MCs. This is in agreement with the favourable prognostic value of PR in feline MCs.^37^ In dogs, luminal (ER+ and/or PR+) MCs showed a late increase in 1‐year CSS, from 76 ± 5% at month 5 to 92 ± 4% at month 12 (+16% gain in CSS in 7 months): the probability for a dog of dying from a luminal MC during the subsequent year became negligible if the dog had survived 1 year. Human breast cancers behave differently, maybe because patients with luminal tumours benefit from 5 to 10 years of hormone therapy: ER‐positive breast cancers are associated with a cancer‐specific mortality peak at 4 years followed by a plateau, whereas ER‐negative breast cancers are associated with a mortality peak at 2 years post‐diagnosis.^57^

As retrospective in nature, this study has limitations. Firstly, the minimal follow‐up period was 2 years, a duration not sufficient enough to identify at which time the MC has no more influence on life expectancy. In human oncology, when the 5‐year relative CSS probability exceeds 95%, one considers that the given cancer does not cause excess mortality anymore; this is achieved in patients who have survived 10 years after diagnosis of a gastric, colorectal, cervical or thyroid cancer, whereas this is not achieved for breast cancer,^33^ probably because of the late mortality peak observed 8 to 10 years after breast cancer diagnosis.^57^, ^58^, ^59^ Secondly, exclusion criteria of this study included the presence of distant metastases at diagnosis, but this parameter could play a role in the CS of dogs and cats, as it does in breast cancer,^43^, ^55^ and it would be interesting to analyse this parameter in a larger cohort. Thirdly, the lack of an age‐matched reference population of dogs and cats without invasive MCs makes it impossible to calculate the relative CS, the best indicator of the impact of invasive MCs on the life and death probabilities of patients. Finally, this retrospective study suffers from inter‐patient heterogeneity in the completeness of cancer staging at diagnosis and during follow‐up: 231/344 dogs (67%) and 219/342 cats (64%) did not have the regional lymph node sampled for histopathology, 119/344 dogs (35%) and 215/342 cats (63%) did not benefit from medical imaging for distant metastasis detection at diagnosis, thus leading to a certain degree of under‐staging. The other bias of retrospective cohorts such as the present one resides in the existence of deaths from unknown causes (65/344 dogs, 19%, and 54/342 cats, 16%), which precludes ideal calculation of CSS probabilities. For these reasons, an external validation of the present results in an independent cohort, with complete staging at diagnosis, regular re‐staging during follow‐up, and precise determination of the cause of deaths in all cases, is required before clinical applications of the present results are considered.

## 5 CONCLUSION

CS appears as an interesting tool to refine the prognosis of dogs and cats with invasive MCs that have survived a few months after mastectomy. Twelve‐month surviving dogs had a 2‐fold decreased risk of dying from cancer in the subsequent year compared with their risk at diagnosis, whereas cats remained at the same high risk of dying from cancer even if they had already survived 1 year. Some prognosticators, such as lymphovascular invasion and nodal stage, durably affected the probability for canine and feline patients of living one further year, even in 12‐month survivors. By comparison, tumour size and histological grade were well associated with early cancer‐related death, but not significantly in surviving patients. This study thus highlights the differences between prognosis establishment at diagnosis, and 1 year later for survivors.

## CONFLICT OF INTEREST

The authors declare no conflict of interest. The funders had no role in study design, data collection and analysis, decision to publish, or preparation of the manuscript.

## ETHICS STATEMENT

The study design was reviewed and approved by the CERVO (Comité d'Ethique en Recherche clinique et épidémiologique Vétérinaire d'Oniris), the local ethical committee of Oniris, Nantes Atlantic College of Veterinary Medicine, Food Science and Engineering, Nantes, France. Written informed consent was obtained from the owners for the participation of their animals in this study.

## Supporting information


**Figure S1** Conditional overall survival (COS) of dogs with invasive mammary carcinomas according to various clinical‐pathological parameters. **A.** Age at diagnosis. At diagnosis as well as in patients that had survived 1 to 12 months post‐diagnosis, a younger age was associated with better overall survival probabilities. **B.** Pathologic tumour size. At diagnosis and in 1‐ to 11‐month surviving dogs, a smaller pathologic tumour size was associated with better COS. **C.** Pathologic nodal stage. At diagnosis as well as in dogs that had survived 1‐12 months, a positive nodal stage was significantly associated with poor overall survival. **D.** Histological stage. Even in dogs that had survived 12 months post‐diagnosis, a stage III mammary carcinoma was associated with lower probabilities of living one further year than a stage I or II mammary carcinoma. **E.** Lymphovascular invasion. At diagnosis but also in surviving dogs, the presence of lymphatic/venous emboli significantly lowered the probabilities for canine patients to be alive 1 year later. **F.** Margin status. An incomplete surgical excision with positive margins durably affected conditional overall survival in dogs with mammary carcinomas. **G.** Ki‐67 index. Mammary carcinomas with a high proliferation index were associated with low probabilities of living one further year, at diagnosis but also in dogs that had survived 1‐12 months. **H.** Immunophenotype. In dogs that had survived 1‐6 months, the fact that their mammary carcinoma was luminal or triple‐negative did not significantly impact conditional survival. However, in long‐term survivors, a luminal mammary carcinoma was associated with better probabilities of living one further year than triple‐negative mammary carcinomas. * P‐value<0.05.

**Figure S2** Conditional specific survival (CSS) of dogs with invasive mammary carcinomas according to various clinical‐pathological parameters. **A.** Age at diagnosis. Diagnosis of MC at an older age was associated with increased risk of dying from cancer, even in 1‐year survivors. **B.** Pathologic tumour size. In dogs that had survived at least 3 months, a larger tumour size was not a negative prognosticator any more. **C.** Pathologic nodal stage. The presence of nodal metastasis was associated with higher probabilities of cancer‐related death, at diagnosis as well as in 1‐12 month survivors. **D.** Histological stage. The negative impact of stage III on conditional specific survival was significant in 1‐ to 12‐month surviving dogs. **E.** Lymphovascular invasion. The probability of dying from cancer in the subsequent year was higher in LVI+ cases than LVI− cases, even in dogs that had already survived 1 year. **F.** Margin status. Positive margins had a negative impact on 1‐year CSS at most time points from 1 to 12 months of post‐diagnosis survival. **G.** Ki‐67 index. In dogs that had survived 11‐12 months, a high proliferation index of their MC was no longer significantly associated with the risk of dying from cancer in the following year. **H.** Immunophenotype. Although luminal and triple‐negative MCs were associated with similar probabilities of cancer‐related death from diagnosis to 6 months post‐diagnosis, the long‐term survivors showed better CSS if their MC was luminal rather than triple‐negative. *P‐value<0.05

**Figure S3** Conditional overall survival (COS) of cats with invasive mammary carcinomas according to various clinical‐pathological parameters. **A.** Pathologic tumour size. The pathologic tumour size affected conditional overall survival only during the first 5 months post‐diagnosis. In cats that had survived at least 6 months, the probability of living one further year was not significantly different between those with smaller and those with larger mammary carcinomas. **B.** Pathologic nodal stage. The presence of nodal metastases had a negative influence on the probability of living one further year at almost any time from diagnosis to 12 months post‐diagnosis. **C.** Histological stage. Even in cats that had already survived 12 months after mammary carcinoma removal, a stage III MC was still associated with a lower probability of living one further year than a stage I or II MC. **D.** Histological grade. Grade III MCs were associated with poorer COS only during the first 2 months post‐diagnosis. **E.** Lymphovascular invasion. The presence of lymphatic/venous emboli was significantly associated with reduced conditional overall survival during the first 6 months; afterwards, the probability of living one further year did not significantly depend on LVI. **F.** Margin status. The negative impact of positive margins on conditional overall survival was only significant during the first 7 months post‐diagnosis. **G.** Immunophenotype. PR‐positive MCs were associated with better conditional survival than PR‐negative MCs, but only significantly during the first 2 months post‐diagnosis. **H.** Ki‐67 index. The proliferation index of feline mammary carcinomas did not significantly influence conditional overall survival. * P‐value<0.05. ** *P* < 0.01.

**Figure S4** Conditional specific survival (CSS) of cats with invasive mammary carcinomas according to various clinical‐pathological parameters. **A.** Pathologic tumour size. The probability for a cat with MC of dying from cancer within the following year was greater if the MC was larger, at diagnosis and during the first 5 months post‐diagnosis; afterwards however, conditional specific survival did not significantly depend on tumour size in cats that had survived at least 6 months. **B.** Pathologic nodal stage. A positive nodal stage durably impacted the probability of dying from cancer within the following year, even in long‐term survivors. **C.** Histological stage. An advanced stage at diagnosis (III) was associated with poorer conditional specific survival. **D.** Histological grade. The effect of histological grade on conditional specific survival was low. **E.** Lymphovascular invasion. Even in cats that had survived 12 months, the presence of lymphovascular invasion remained a pejorative factor associated with higher probabilities of dying from cancer during the following year. **F.** Margins status. An incomplete surgical excision with positive margins was associated with higher probabilities of dying from cancer, at diagnosis, but also in cats that had survived 1‐6 months. **G.** Immunophenotype. PR‐positive mammary carcinomas were durably associated with a lower risk of dying from cancer during the following year than PR‐negative MCs. **H.** Ki‐67 index. The proliferation index of feline MCs did not significantly influence the probabilities of dying from cancer during the following year. * P‐value<0.05. ** *P* < 0.01.

**Appendix** **S1:** Supporting information

## Data Availability

The data that support the findings of this study are available on request from the corresponding author.
