# Supplementary figures and images for: One‐year conditional survival of dogs and cats with invasive mammary carcinomas: A concept inspired from human breast cancer
Source: Vet Comp Oncol. 2020 Sep 28;19(1):140–51. doi: 10.1111/vco.12655 (PMC7891631; doi:10.1111/vco.12655)

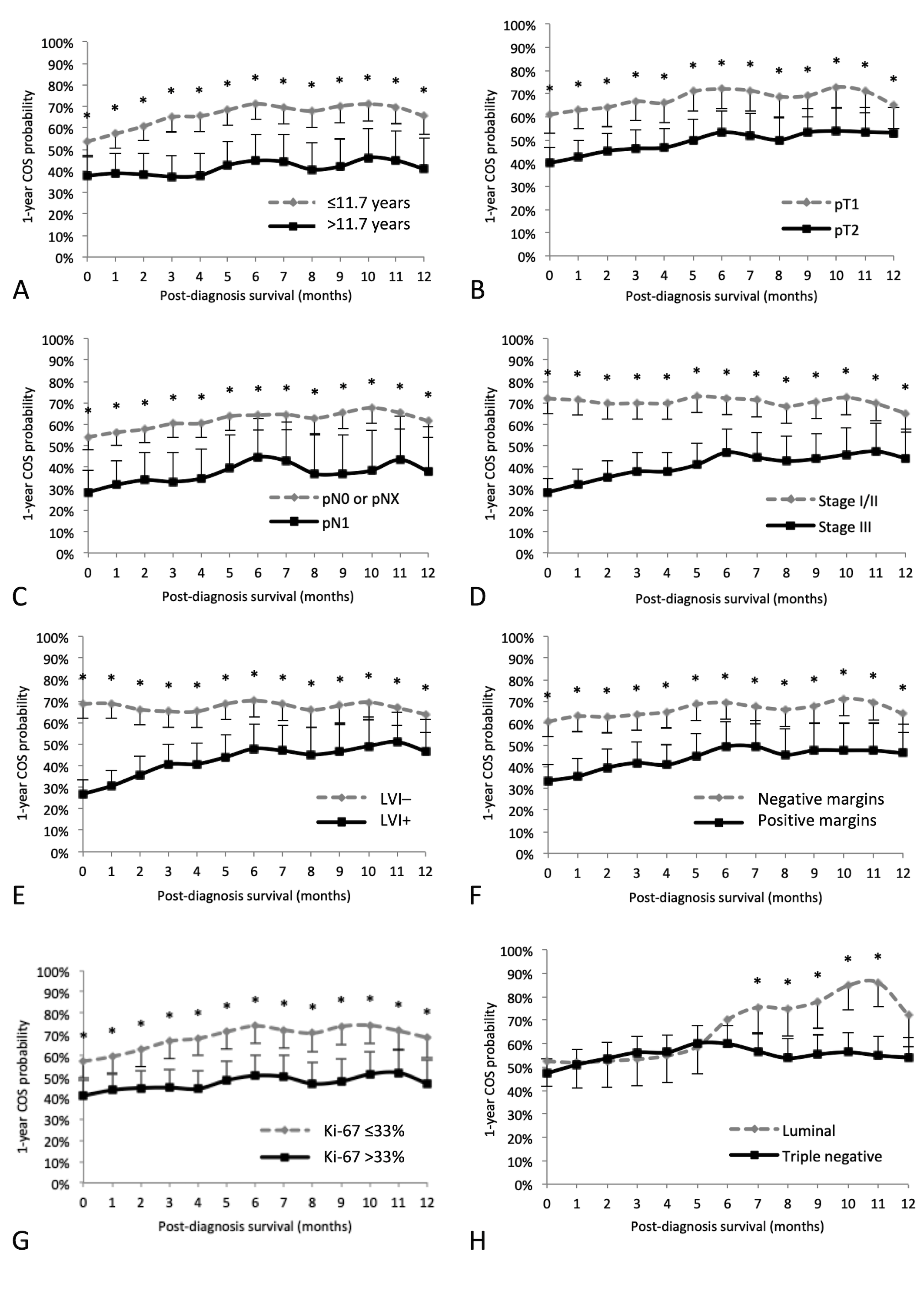

Supplement: Supplementary file 1 — Figure S1 Conditional overall survival (COS) of dogs with invasive mammary carcinomas according to various clinical‐pathological parameters. A. Age at diagnosis. At diagnosis as well as in patients that had survived 1 to 12 months post‐diagnosis, a younger age was associated with better overall survival probabilities. B. Pathologic tumour size. At diagnosis and in 1‐ to 11‐month surviving dogs, a smaller pathologic tumour size was associated with better COS. C. Pathologic nodal stage. At diagnosis as well as in dogs that had survived 1‐12 months, a positive nodal stage was significantly associated with poor overall survival. D. Histological stage. Even in dogs that had survived 12 months post‐diagnosis, a stage III mammary carcinoma was associated with lower probabilities of living one further year than a stage I or II mammary carcinoma. E. Lymphovascular invasion. At diagnosis but also in surviving dogs, the presence of lymphatic/venous emboli significantly lowered the probabilities for canine patients to be alive 1 year later. F. Margin status. An incomplete surgical excision with positive margins durably affected conditional overall survival in dogs with mammary carcinomas. G. Ki‐67 index. Mammary carcinomas with a high proliferation index were associated with low probabilities of living one further year, at diagnosis but also in dogs that had survived 1‐12 months. H. Immunophenotype. In dogs that had survived 1‐6 months, the fact that their mammary carcinoma was luminal or triple‐negative did not significantly impact conditional survival. However, in long‐term survivors, a luminal mammary carcinoma was associated with better probabilities of living one further year than triple‐negative mammary carcinomas. * P‐value<0.05. [file VCO-19-140-s001.tif]

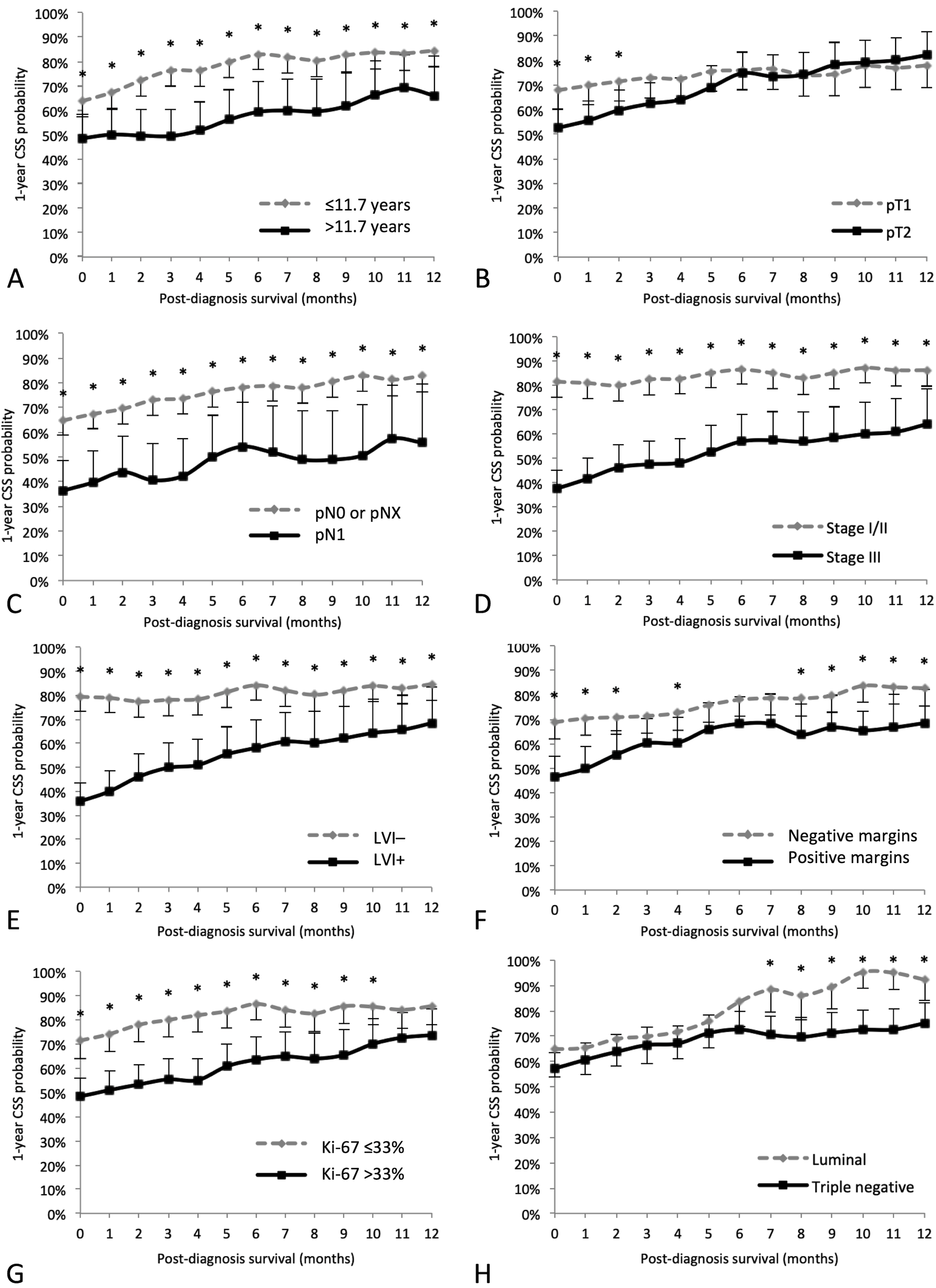

Supplement: Supplementary file 2 — Figure S2 Conditional specific survival (CSS) of dogs with invasive mammary carcinomas according to various clinical‐pathological parameters. A. Age at diagnosis. Diagnosis of MC at an older age was associated with increased risk of dying from cancer, even in 1‐year survivors. B. Pathologic tumour size. In dogs that had survived at least 3 months, a larger tumour size was not a negative prognosticator any more. C. Pathologic nodal stage. The presence of nodal metastasis was associated with higher probabilities of cancer‐related death, at diagnosis as well as in 1‐12 month survivors. D. Histological stage. The negative impact of stage III on conditional specific survival was significant in 1‐ to 12‐month surviving dogs. E. Lymphovascular invasion. The probability of dying from cancer in the subsequent year was higher in LVI+ cases than LVI− cases, even in dogs that had already survived 1 year. F. Margin status. Positive margins had a negative impact on 1‐year CSS at most time points from 1 to 12 months of post‐diagnosis survival. G. Ki‐67 index. In dogs that had survived 11‐12 months, a high proliferation index of their MC was no longer significantly associated with the risk of dying from cancer in the following year. H. Immunophenotype. Although luminal and triple‐negative MCs were associated with similar probabilities of cancer‐related death from diagnosis to 6 months post‐diagnosis, the long‐term survivors showed better CSS if their MC was luminal rather than triple‐negative. *P‐value<0.05 [file VCO-19-140-s002.tif]

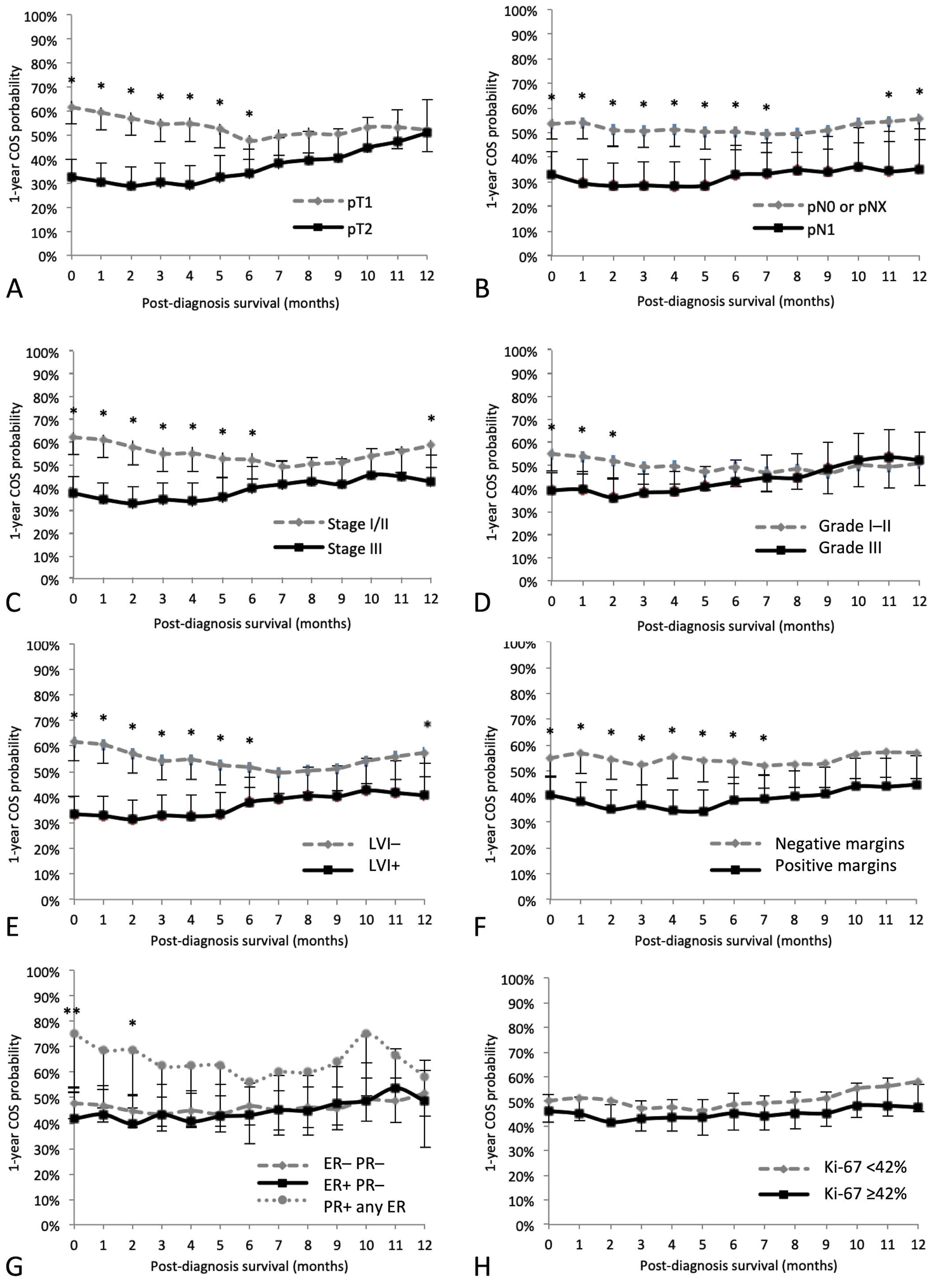

Supplement: Supplementary file 3 — Figure S3 Conditional overall survival (COS) of cats with invasive mammary carcinomas according to various clinical‐pathological parameters. A. Pathologic tumour size. The pathologic tumour size affected conditional overall survival only during the first 5 months post‐diagnosis. In cats that had survived at least 6 months, the probability of living one further year was not significantly different between those with smaller and those with larger mammary carcinomas. B. Pathologic nodal stage. The presence of nodal metastases had a negative influence on the probability of living one further year at almost any time from diagnosis to 12 months post‐diagnosis. C. Histological stage. Even in cats that had already survived 12 months after mammary carcinoma removal, a stage III MC was still associated with a lower probability of living one further year than a stage I or II MC. D. Histological grade. Grade III MCs were associated with poorer COS only during the first 2 months post‐diagnosis. E. Lymphovascular invasion. The presence of lymphatic/venous emboli was significantly associated with reduced conditional overall survival during the first 6 months; afterwards, the probability of living one further year did not significantly depend on LVI. F. Margin status. The negative impact of positive margins on conditional overall survival was only significant during the first 7 months post‐diagnosis. G. Immunophenotype. PR‐positive MCs were associated with better conditional survival than PR‐negative MCs, but only significantly during the first 2 months post‐diagnosis. H. Ki‐67 index. The proliferation index of feline mammary carcinomas did not significantly influence conditional overall survival. * P‐value<0.05. ** P < 0.01. [file VCO-19-140-s003.tif]

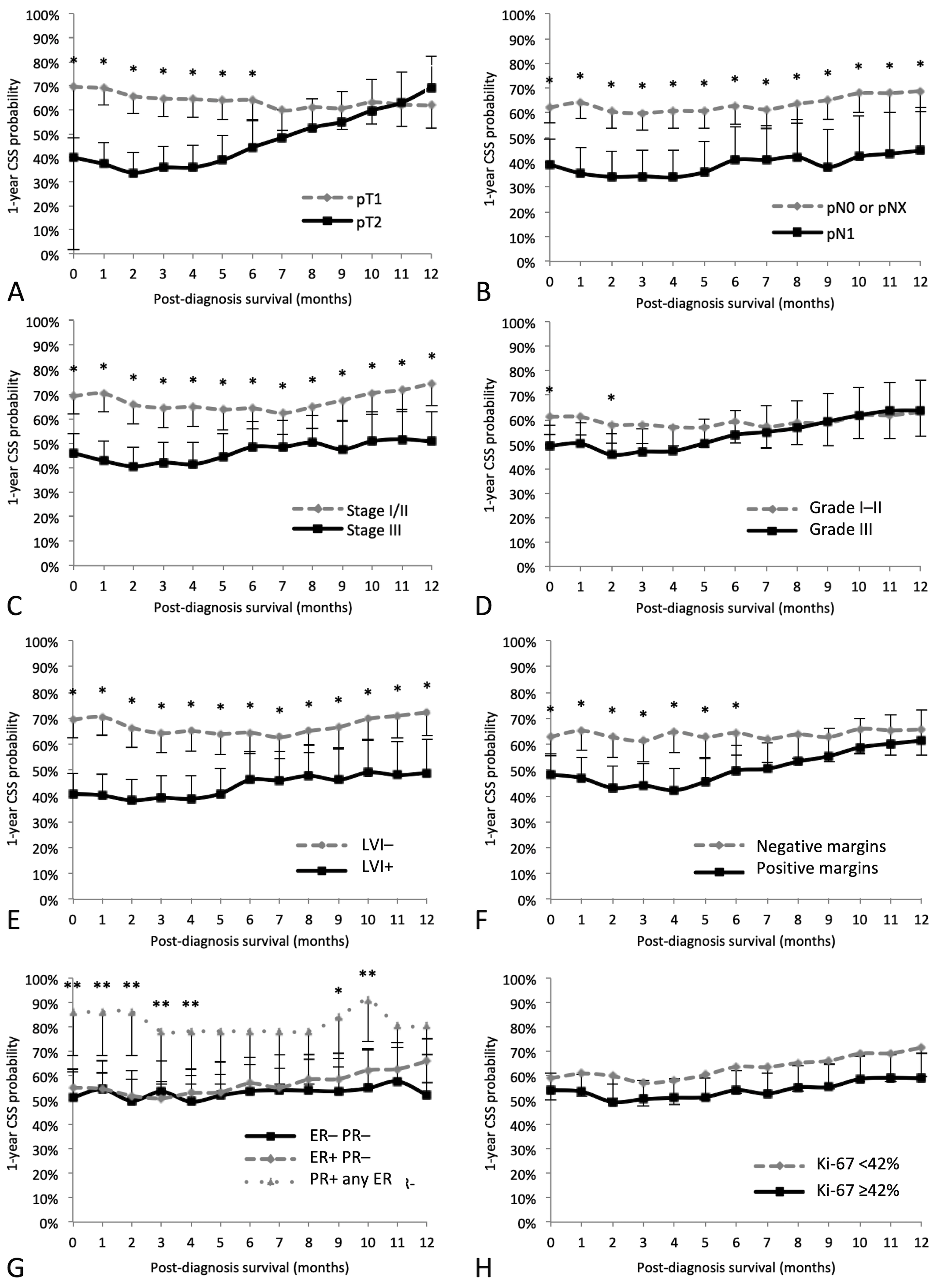

Supplement: Supplementary file 4 — Figure S4 Conditional specific survival (CSS) of cats with invasive mammary carcinomas according to various clinical‐pathological parameters. A. Pathologic tumour size. The probability for a cat with MC of dying from cancer within the following year was greater if the MC was larger, at diagnosis and during the first 5 months post‐diagnosis; afterwards however, conditional specific survival did not significantly depend on tumour size in cats that had survived at least 6 months. B. Pathologic nodal stage. A positive nodal stage durably impacted the probability of dying from cancer within the following year, even in long‐term survivors. C. Histological stage. An advanced stage at diagnosis (III) was associated with poorer conditional specific survival. D. Histological grade. The effect of histological grade on conditional specific survival was low. E. Lymphovascular invasion. Even in cats that had survived 12 months, the presence of lymphovascular invasion remained a pejorative factor associated with higher probabilities of dying from cancer during the following year. F. Margins status. An incomplete surgical excision with positive margins was associated with higher probabilities of dying from cancer, at diagnosis, but also in cats that had survived 1‐6 months. G. Immunophenotype. PR‐positive mammary carcinomas were durably associated with a lower risk of dying from cancer during the following year than PR‐negative MCs. H. Ki‐67 index. The proliferation index of feline MCs did not significantly influence the probabilities of dying from cancer during the following year. * P‐value<0.05. ** P < 0.01. [file VCO-19-140-s004.tif]
