# Supplementary material for: One‐year conditional survival of dogs and cats with invasive mammary carcinomas: A concept inspired from human breast cancer
Source: Vet Comp Oncol. 2020 Sep 28;19(1):140–51. doi: 10.1111/vco.12655 (PMC7891631; doi:10.1111/vco.12655)

**One-year conditional survival of dogs and cats with invasive mammary carcinomas: a concept inspired from human breast cancer.**

**Supplementary information**

**Follow-up of female dogs with mammary carcinoma**

The median time to locoregional recurrence (LRR) was 26.4 months; the LRR probability was 34% at 1 year, and 48% at 2 years post-diagnosis. At the end of the follow-up period, 76 dogs (22%) had experienced tumor recurrence at the site of prior mastectomy, 56 dogs (16%) a new primary mammary tumor, and 18 dogs (5%) more than one locoregional event. A second surgery was performed in 12/76 dogs (16%) for local recurrence, in 29/56 dogs (52%) for a new primary mammary tumor, and in 4/18 dogs (22%) for multifocal locoregional relapse, for a total of 45 second surgeries (30%) in the 150 dogs that had locoregional relapse. Of note, the second surgery for LRR had a very significant impact on OS and SS; unexpectedly, overall survival was even longer in dogs, which benefited from a second surgery for LRR, than in dogs with no reported locoregional relapses:

Survival of female dogs with mammary carcinoma according to LRR with or without second surgery.

| **Overall survival** | Hazard Ratio HR | 95% CI | p | Median OS |
| --- | --- | --- | --- | --- |
| No LRR (N=194) | 1.00 (reference) | – | – | 13.1 months |
| LRR, untreated (N=105) | 1.41 | 1.11 – 1.81 | 0.0061 | 5.7 months |
| LRR + second surgery (N=45) | 0.64 | 0.44 – 0.92 | 0.0161 | 23.4 months |

| **Cancer-specific survival** | Hazard Ratio HR | 95% CI | p | Median SS |
| --- | --- | --- | --- | --- |
| No LRR (N=194) | 1.00 (reference) | – | – | 31.5 months |
| LRR, untreated (N=105) | 2.51 | 1.85 – 3.42 | <0.0001 | 7.0 months |
| LRR + second surgery (N=45) | 0.87 | 0.53 – 1.42 | NS | 44.4 months |

The median time to distant metastasis was unreached. Distant metastasis was present in 16% of dogs at 1 year post-diagnosis, 23% at two years post-diagnosis, and 29% (101/344) at the end of follow-up. Of note, the occurrence of distant metastasis was significantly associated with poor overall survival (Hazard Ratio HR=1.67, 95% CI 1.28–2.18, p<0.0001, median OS of 14.6 months for M0 cases and 7.9 months for M1 cases), and poor cancer-specific survival (HR=3.19, 95% CI 2.28–4.47, p<0.0001, median SS of 56.4 months for M0 cases, and 8.1 months for M1 cases).

Among the 304 dogs (88%) that died during the follow-up period, 58 (19%) died from intercurrent diseases, 65 (21%) died from unknown cause, and 181 (60%) deaths were due to cancer, including

- 24 (13%) attributable to inoperable locoregional progression (inflammatory mammary carcinoma, simultaneous distant metastases),
- 36 (20%) attributable to locoregional relapse that owners were not willing to treat,
- 55 (30%) attributable to distant metastases confirmed by medical imaging (in the absence of locoregional recurrence),
- 39 (22%) attributable to suspected distant metastases based on cough and/or dyspnea (in the absence of locoregional recurrence), in patients that had evidence of metastatic spread of their mammary carcinoma through the lymphatic route (LVI+ and/or pN+, 23/39 dogs), or had a serohemorrhagic pleural transudate suggestive of pleural/pulmonary metastases (without confirmation by thoracic radiographs, but also without intercurrent respiratory diseases, 16/39 dogs),
- and 27 (15%) attributable to terminal cancer: anorexia and/or cachexia, in the absence of intercurrent disease, in patients with lymphatic spread of their mammary carcinoma (19/27 dogs), or patients which already presented anorexia or cachexia at diagnosis of their mammary carcinoma and died shortly thereafter (8/27 dogs).

**Follow-up of female cats with mammary carcinoma**

The median time to locoregional recurrence was 16.1 months; the LRR probability was 41% at 1 year, and 58% at 2 years post-diagnosis. At the end of the follow-up period, 78 cats (23%) had experienced tumor recurrence at the site of prior mastectomy, 76 cats (22%) a new primary mammary tumor, and 20 cats (6%) more than one locoregional event. A second surgery was performed in 20/78 cats (26%) for local recurrence, in 23/76 cats (30%) for a new primary mammary tumor, and in 9/20 cats (45%) for multifocal locoregional relapse, for a total of 52 second surgeries (30%) in the 174 cats that had locoregional relapse. The second surgery for LRR had a very significant favorable impact on OS, while leaving the locoregional relapse untreated was significantly associated with a poorer SS.

Survival of female cats with mammary carcinoma according to LRR with or without second surgery.

| **Overall survival** | Hazard Ratio HR | 95% CI | p | Median OS |
| --- | --- | --- | --- | --- |
| No LRR (N=168) | 1.00 (reference) | – | – | 8.2 months |
| LRR, untreated (N=122) | 0.96 | 0.78 – 1.27 | NS | 9.5 months |
| LRR + second surgery (N=52) | 0.50 | 0.35 – 0.71 | 0.0001 | 20.6 months |

| **Cancer-specific survival** | Hazard Ratio HR | 95% CI | p | Median SS |
| --- | --- | --- | --- | --- |
| No LRR (N=168) | 1.00 (reference) | – | – | 14.7 months |
| LRR, untreated (N=122) | 1.62 | 1.22 – 2.15 | 0.0010 | 11.3 months |
| LRR + second surgery (N=52) | 0.75 | 0.51 – 1.12 | NS | 25.2 months |

The median time to distant metastasis was unreached. Distant metastasis was present in 17% of cats at 1 year post-diagnosis, 26% at two years post-diagnosis, and 34% (117/342) at the end of follow-up. As in dogs, the occurrence of distant metastasis was significantly associated with poor overall survival (HR=1.73, 95% CI 1.34–2.23, p<0.0001, median OS of 13.3 months for M0 cases and 8.1 months for M1 cases) and poor cancer-specific survival (HR=2.32, 95% CI 1.73–3.12, p<0.0001, median SS of 22.9 months for M0 cases and 8.5 months for M1 cases).

Among the 305 cats (89%) that died during the follow-up, 25 (8%) died from intercurrent diseases, 54 (18%) died from unknown cause, and 226 (74%) deaths were due to cancer, including

- 23 (10%) attributable to inoperable locoregional progression (very large / diffuse mammary carcinoma, simultaneous distant metastases),
- 63 (28%) attributable to locoregional relapse that owners were not willing to treat,
- 69 (30%) attributable to distant metastases confirmed by medical imaging (in the absence of locoregional recurrence),
- 40 (18%) attributable to suspected distant metastases based on cough and/or dyspnea (in the absence of locoregional recurrence), in patients that had evidence of metastatic spread of their mammary carcinoma through the lymphatic route (LVI+ and/or pN+, 28/40 cats), or had a serohemorrhagic pleural transudate suggestive of pleural/pulmonary metastases (without confirmation by thoracic radiographs, but also without intercurrent respiratory diseases, 12/40 cats),
- and 31 (14%) attributable to terminal cancer: anorexia and/or cachexia, in the absence of intercurrent disease, in patients with lymphatic spread of their mammary carcinoma (23/31 cats), or patients which already presented anorexia or cachexia at diagnosis of their mammary carcinoma and died shortly thereafter (8/31 dogs).

**Supplementary Table 1**. Primary antibodies and immunohistochemical protocols.

|  | | **p63** | **Pancytokeratin** | **LMO2 (cat)** | **ERα** | **PR (dog)** | **PR (cat)** | **HER2** | **Ki-67** |
| --- | --- | --- | --- | --- | --- | --- | --- | --- | --- |
| Primary antibody | Clone | BC4A4 | AE1/AE3 | SP51 | C311 | 1E2 | 10A9 | 4B5 | MIB1 |
|  | Type | Mouse monoclonal | Mouse  monoclonal | Rabbit  monoclonal | Mouse  monoclonal | Rabbit  monoclonal | Mouse  monoclonal | Rabbit  monoclonal | Mouse  monoclonal |
|  | Supplier reference | Abcam ab735 | Dako  M3515 | Spring  M351 | Santa Cruz Biotechnology sc787 | Ventana Medical Systems  790-4296 | Interchim K42546M | Ventana Medical Systems 790-2991 | Dako  M7240 |
|  | Dilution | 1:50 | 1:100 | 1:150 | 1:50 | prediluted | 1:50 | prediluted | 1:50 |
|  | Incubation time | 32 min | 44 min | 92 min | 44 min | 32 min | 100 min | 8 min | 32 min |
| Antigen Retrieval |  | 30 min  (95°C-CC1) | 8 min  (95°C-CC1)  Protease 8 min | 48 min  (95°C-CC1) | None | 90 min  (95°C-CC1) | 60 min  (95°C-CC1) | 30 min  (95°C-CC1) | 60 min  (95°C-CC1) |
| Detection system |  | Iview^a^ | Iview^a^ | Optiview^b^ | Iview^a^ | Iview^a^ | Iview^a^ | Ultraview^c^ | Iview^a^ |

All dilutions were performed in a commercially available antibody diluent (Ventana Medical Systems #251-018).

^a^ Iview DAB detection kit: indirect biotin streptavidin DAB detection system (Ventana Medical Systems #760-091).

^b^ Optiview DAB IHC detection kit: biotin-free multimer-based detection system with 3,3'-diaminobenzidine tetrahydrochloride (DAB) as chromogen (Ventana Medical Systems #760-700).

^c^ Ultraview universal DAB detection kit: indirect biotin-free system for detecting mouse IgG, mouse IgM and rabbit primary antibodies with DAB detection system (Ventana Medical Systems #760-500).

CC1 (Cell Conditioning solution 1): basic unmasking buffer (Ventana Medical Systems #950-124).

Protease-1 (Ventana Medical Systems #760-2018).

**Supplementary Table 2.** Comparison between dogs which died before and after 1 year post-diagnosis.

| Parameter |  | Total  N=344 | Death within 1 year post-diagnosis  N=177 | Survival >1 year  N=167 | p-value |
| --- | --- | --- | --- | --- | --- |
| Age (years) | mean ± SD | 11.0 ± 2.1 | 11.3 ± 2.1 | 10.6 ± 2.1 | **0.003** |
| Neutering status | Intact female | 248 (72%) | 123 (69%) | 125 (75%) | NS |
|  | Neutered female | 96 (28%) | 54 (31%) | 42 (25%) |  |
| Contraception | Yes | 20 (6%) | 10 (5%) | 10 (6%) | NS |
|  | No or Unknown | 324 (94%) | 167 (95%) | 157 (94%) |  |
| Multicentricity | Yes | 52 (15%) | 35 (20%) | 17 (10%) | **0.0197** |
|  | No | 292 (85%) | 142 (80%) | 150 (90%) |  |
| Pathologic tumor size (mm) | mean ± SD (a) | 17.8 ± 6.9 | 18.8 ± 7.6 | 17.1 ± 6.3 | 0.067 |
|  | pT1 | 160 (47%) | 65 (37%) | 95 (57%) | **0.0002** |
|  | pT2 | 184 (53%) | 112 (63%) | 72 (43%) |  |
| Pathological nodal stage | pN1 | 74 (22%) | 53 (30%) | 21 (12%) | **<0.0001** |
|  | pN0 | 39 (11%) | 11 (6%) | 28 (17%) |  |
|  | pNX | 231 (67%) | 113 (64%) | 118 (71%) |  |
| Histological type | Complex | 31 (9%) | 11 (6%) | 20 (12%) | **0.0025** |
|  | Anaplastic | 21 (6%) | 18 (10%) | 3 (2%) |  |
|  | Solid | 100 (29%) | 49 (28%) | 51 (30%) |  |
|  | Tubulopapillary | 173 (50%) | 88 (50%) | 85 (51%) |  |
|  | Inflammatory | 5 (2%) | 5 (3%) | 0 (0%) |  |
|  | Squamous cell | 14 (4%) | 6 (3%) | 8 (5%) |  |
| Histological grade | Grade I | 19 (6%) | 3 (2%) | 16 (10%) | **<0.0001** |
|  | Grade II | 104 (30%) | 44 (25%) | 60 (36%) |  |
|  | Grade III | 221 (64%) | 130 (73%) | 91 (54%) |  |
| Lymphovascular invasion | LVI+ | 167 (49%) | 122 (69%) | 45 (27%) | **<0.0001** |
|  | LVI– | 177 (51%) | 55 (31%) | 122 (73%) |  |
| Histological stage | Stage I | 81 (24%) | 14 (8%) | 67 (40%) | **<0.0001** |
|  | Stage II | 79 (23%) | 31 (18%) | 48 (29%) |  |
|  | Stage IIIA | 79 (23%) | 51 (29%) | 28 (17%) |  |
|  | Stage IIIB | 105 (30%) | 81 (45%) | 24 (14%) |  |
| Margins | Negative | 189 (55%) | 74 (42%) | 115 (69%) | **<0.0001** |
|  | Positive | 155 (45%) | 103 (58%) | 52 (31%) |  |
| Dermal infiltration | Yes | 117 (34%) | 66 (37%) | 51 (30%) | NS |
|  | No | 227 (66%) | 111 (63%) | 116 (70%) |  |
| Cutaneous ulceration | Yes | 48 (14%) | 29 (16%) | 19 (11%) | NS |
|  | No | 296 (86%) | 148 (84%) | 148 (89%) |  |
| Muscle infiltration | Yes | 65 (19%) | 43 (24%) | 22 (13%) | **0.0126** |
|  | No | 279 (81%) | 134 (76%) | 145 (87%) |  |
| Central necrosis | Yes | 256 (74%) | 126 (71%) | 130 (78%) | NS |
|  | No | 88 (26%) | 51 (29%) | 37 (22%) |  |
| Inflammation | Moderate to severe | 166 (48%) | 107 (55%) | 59 (35%) | **<0.0001** |
|  | Absent to mild | 178 (52%) | 70 (45%) | 108 (65%) |  |
| ER expression | ER <10% | 288 (84%) | 150 (85%) | 138 (83%) | NS |
|  | ER ≥10% | 56 (16%) | 27 (15%) | 29 (17%) |  |
| PR expression | PR <10% | 304 (88%) | 158 (89%) | 146 (87%) | NS |
|  | PR ≥10% | 40 (12%) | 19 (11%) | 21 (13%) |  |
| HER2 expression | Score 0 | 243 (71%) | 127 (72%) | 116 (69%) | NS |
|  | Score 1+ | 75 (22%) | 39 (22%) | 36 (22%) |  |
|  | Score 2+ | 26 (7%) | 11 (6%) | 15 (9%) |  |
| Immunophenotype | Luminal | 82 (24%) | 39 (22%) | 43 (26%) | NS |
|  | Triple Negative | 262 (76%) | 138 (78%) | 124 (74%) |  |
| Ki-67 index | Ki-67 ≤33% | 161 (46%) | 69 (39%) | 92 (55%) | **0.0039** |
|  | Ki-67 >33% | 183 (54%) | 108 (61%) | 75 (45%) |  |

(a) The pathologic tumor size in mm was available in 227 dogs only (96 dead within 1 year post-diagnosis, 131 that survived at least one year), because some very large tumors and some tumors with positive margins were not precisely measurable on tissue sections. The cases for which pT was not precisely measurable in millimeters, fall into the pT2 category (pT >20 mm).

**Supplementary Table 3.** Comparison between cats which died before and after 1 year post-diagnosis.

| Parameter |  | Total  N=342 | Death within 1 year post-diagnosis  N=175 | Survival >1 year  N=167 | p-value |
| --- | --- | --- | --- | --- | --- |
| Age (years) | mean ± SD | 11.2 ± 2.7 | 11.2 ± 2.6 | 11.3 ± 2.9 | NS |
| Neutering status | Intact female | 179 (52%) | 98 (56%) | 81 (49%) | NS |
|  | Neutered female | 163 (48%) | 77 (44%) | 86 (51%) |  |
| Contraception | Yes | 139 (41%) | 72 (41%) | 67 (40%) | NS |
|  | No or Unknown | 203 (59%) | 103 (59%) | 100 (60%) |  |
| Multicentricity | Yes | 49 (14%) | 28 (16%) | 21 (13%) | NS |
|  | No | 293 (86%) | 147 (84%) | 146 (87%) |  |
| Pathologic tumor size (mm) | mean ± SD (a) | 17.9 ± 7.4 | 20.1 ± 7.7 | 15.7 ± 6.5 | **<0.001** |
|  | pT1 | 188 (56%) | 73 (42%) | 115 (70%) | **<0.0001** |
|  | pT2 | 151 (44%) | 102 (58%) | 49 (30%) |  |
| Pathological nodal stage | pN1 | 97 (28%) | 63 (36%) | 34 (21%) | **<0.0001** |
|  | pN0 | 26 (8%) | 4 (2%) | 22 (13%) |  |
|  | pNX | 219 (64%) | 108 (62%) | 111 (66%) |  |
| Histological type | Cribriform | 171 (50%) | 83 (47%) | 88 (53%) | NS |
|  | Mucinous | 37 (11%) | 18 (10%) | 19 (11%) |  |
|  | Solid | 67 (19%) | 36 (21%) | 31 (19%) |  |
|  | Tubulopapillary | 56 (17%) | 29 (17%) | 27 (16%) |  |
|  | Other (b) | 11 (3%) | 9 (5%) | 2 (1%) |  |
| Histological grade | Grade I | 10 (3%) | 4 (2%) | 6 (4%) | **0.0168** |
|  | Grade II | 172 (50%) | 76 (44%) | 96 (57%) |  |
|  | Grade III | 160 (47%) | 95 (54%) | 65 (39%) |  |
| Lymphovascular invasion | LVI+ | 168 (49%) | 109 (62%) | 59 (35%) | **<0.0001** |
|  | LVI– | 174 (51%) | 66 (38%) | 108 (65%) |  |
| Histological stage | Stage I | 104 (30%) | 33 (19%) | 71 (43%) | **<0.0001** |
|  | Stage II | 54 (16%) | 29 (17%) | 25 (15%) |  |
|  | Stage IIIA | 84 (25%) | 40 (23%) | 44 (26%) |  |
|  | Stage IIIB | 100 (29%) | 73 (41%) | 27 (16%) |  |
| Margins | Negative | 169 (49%) | 76 (43%) | 93 (56%) | **0.0309** |
|  | Positive | 173 (51%) | 99 (57%) | 74 (44%) |  |
| Dermal infiltration | Yes | 207 (61%) | 128 (73%) | 79 (47%) | **<0.0001** |
|  | No | 135 (39%) | 47 (27%) | 88 (53%) |  |
| Cutaneous ulceration | Yes | 76 (22%) | 51 (29%) | 25 (15%) | **0.0025** |
|  | No | 266 (78%) | 124 (71%) | 142 (85%) |  |
| Muscle infiltration | Yes | 198 (58%) | 104 (59%) | 94 (56%) | NS |
|  | No | 144 (42%) | 71 (41%) | 73 (44%) |  |
| Central necrosis | Yes | 307 (90%) | 161 (92%) | 146 (87%) | NS |
|  | No | 35 (10%) | 14 (8%) | 21 (13%) |  |
| Inflammation | Moderate to severe | 176 (51%) | 97 (55%) | 79 (47%) | NS |
|  | Absent to mild | 166 (49%) | 78 (45%) | 88 (53%) |  |
| ER expression | ER <10% | 252 (74%) | 127 (73%) | 125 (75%) | NS |
|  | ER ≥10% | 90 (26%) | 48 (27%) | 42 (25%) |  |
| PR expression | PR <10% | 326 (95%) | 171 (98%) | 155 (93%) | 0.0589 |
|  | PR ≥10% | 16 (5%) | 4 (2%) | 12 (7%) |  |
| HER2 expression | Score 0 | 200 (58%) | 103 (59%) | 97 (58%) | NS |
|  | Score 1+ | 113 (33%) | 58 (33%) | 55 (33%) |  |
|  | Score 2+ | 29 (9%) | 14 (8%) | 15 (9%) |  |
| Immunophenotype | Luminal | 100 (29%) | 51 (29%) | 49 (29%) | NS |
|  | Triple Negative | 242 (71%) | 124 (71%) | 118 (71%) |  |
| Ki-67 index | Ki-67 <42% | 123 (36%) | 61 (35%) | 62 (37%) | NS |
|  | Ki-67 ≥42% | 219 (64%) | 114 (65%) | 105 (63%) |  |

(a) The pathologic tumor size in mm was available in 288 cats only (140 dead within 1 year post-diagnosis, 148 that survived at least one year), because some very large tumors and some tumors with positive margins were not precisely measurable on tissue sections. The cases for which pT was not precisely measurable in millimeters, fall into the pT2 category (pT >20 mm).

(b) Other histological types: adenosquamous, anaplastic, and squamous cell carcinomas. Note that “tubulopapillary” mammary carcinomas refer to tubular, papillary, and tubulopapillary mammary carcinomas.

**Supplementary Figure 1.** Conditional overall survival (COS) of dogs with invasive mammary carcinomas according to various clinical-pathological parameters. **A.** Age at diagnosis. At diagnosis as well as in patients that had survived 1–12 months post-diagnosis, a younger age was associated with better overall survival probabilities. **B.** Pathologic tumor size. At diagnosis and in 1- to 11-month surviving dogs, a smaller pathologic tumor size was associated with better COS. **C.** Pathologic nodal stage. At diagnosis as well as in dogs that had survived 1–12 months, a positive nodal stage was significantly associated with poor overall survival. **D.** Histological stage. Even in dogs that had survived 12 months post-diagnosis, a stage III mammary carcinoma was associated with lower probabilities of living one further year than a stage I or II mammary carcinoma. **E.** Lymphovascular invasion. At diagnosis but also in surviving dogs, the presence of lymphatic/venous emboli significantly lowered the probabilities for canine patients to be alive one year later. **F.** Margin status. An incomplete surgical excision with positive margins durably affected conditional overall survival in dogs with mammary carcinomas. **G.** Ki-67 index. Mammary carcinomas with a high proliferation index were associated with low probabilities of living one further year, at diagnosis but also in dogs that had survived 1–12 months. **H.** Immunophenotype. In dogs that had survived 1–6 months, the fact that their mammary carcinoma was luminal or triple-negative did not significantly impact conditional survival. However, in long-term survivors, a luminal mammary carcinoma was associated with better probabilities of living one further year than triple-negative mammary carcinomas. * p-value<0.05.

**Supplementary Figure 2.** Conditional specific survival (CSS) of dogs with invasive mammary carcinomas according to various clinical-pathological parameters. **A.** Age at diagnosis. Diagnosis of MC at an older age was associated with increased risk of dying from cancer, even in one-year survivors. **B.** Pathologic tumor size. In dogs that had survived at least 3 months, a larger tumor size was not a negative prognosticator any more. **C.** Pathologic nodal stage. The presence of nodal metastasis was associated with higher probabilities of cancer-related death, at diagnosis as well as in 1–12 month survivors. **D.** Histological stage. The negative impact of stage III on conditional specific survival was significant in 1- to 12-month surviving dogs. **E.** Lymphovascular invasion. The probability of dying from cancer in the subsequent year was higher in LVI+ cases than LVI– cases, even in dogs that had already survived one year. **F.** Margin status. Positive margins had a negative impact on one-year CSS at most time points from 1 to 12 months of post-diagnosis survival. **G.** Ki-67 index. In dogs that had survived 11-12 months, a high proliferation index of their MC was no longer significantly associated with the risk of dying from cancer in the following year. **H.** Immunophenotype. Although luminal and triple-negative MCs were associated with similar probabilities of cancer-related death from diagnosis to 6 months post-diagnosis, the long-term survivors showed better CSS if their MC was luminal rather than triple-negative. *p-value<0.05

**Supplementary Figure 3.** Conditional overall survival (COS) of cats with invasive mammary carcinomas according to various clinical-pathological parameters. **A.** Pathologic tumor size. The pathologic tumor size affected conditional overall survival only during the first 5 months post-diagnosis. In cats that had survived at least 6 months, the probability of living one further year was not significantly different between those with smaller and those with larger mammary carcinomas. **B.** Pathologic nodal stage. The presence of nodal metastases had a negative influence on the probability of living one further year at almost any time from diagnosis to 12 months post-diagnosis. **C.** Histological stage. Even in cats that had already survived 12 months after mammary carcinoma removal, a stage III MC was still associated with a lower probability of living one further year than a stage I or II MC. **D.** Histological grade. Grade III MCs were associated with poorer COS only during the first 2 months post-diagnosis. **E.** Lymphovascular invasion. The presence of lymphatic/venous emboli was significantly associated with reduced conditional overall survival during the first 6 months; afterwards, the probability of living one further year did not significantly depend on LVI. **F.** Margin status. The negative impact of positive margins on conditional overall survival was only significant during the first 7 months post-diagnosis. **G.** Immunophenotype. PR-positive MCs were associated with better conditional survival than PR-negative MCs, but only significantly during the first 2 months post-diagnosis. **H.** Ki-67 index. The proliferation index of feline mammary carcinomas did not significantly influence conditional overall survival. * p-value<0.05. ** p<0.01.

**Supplementary Figure 4.** Conditional specific survival (CSS) of cats with invasive mammary carcinomas according to various clinical-pathological parameters. **A.** Pathologic tumor size. The probability for a cat with MC of dying from cancer within the following year was greater if the MC was larger, at diagnosis and during the first 5 months post-diagnosis; afterwards however, conditional specific survival did not significantly depend on tumor size in cats that had survived at least 6 months. **B.** Pathologic nodal stage. A positive nodal stage durably impacted the probability of dying from cancer within the following year, even in long-term survivors. **C.** Histological stage. An advanced stage at diagnosis (III) was associated with poorer conditional specific survival. **D.** Histological grade. The effect of histological grade on conditional specific survival was low. **E.** Lymphovascular invasion. Even in cats that had survived 12 months, the presence of lymphovascular invasion remained a pejorative factor associated with higher probabilities of dying from cancer during the following year. **F.** Margins status. An incomplete surgical excision with positive margins was associated with higher probabilities of dying from cancer, at diagnosis, but also in cats that had survived 1–6 months. **G.** Immunophenotype. PR-positive mammary carcinomas were durably associated with a lower risk of dying from cancer during the following year than PR-negative MCs. **H.** Ki-67 index. The proliferation index of feline MCs did not significantly influence the probabilities of dying from cancer during the following year. * p-value<0.05. ** p<0.01.

**Supplementary Figure 1.**

**
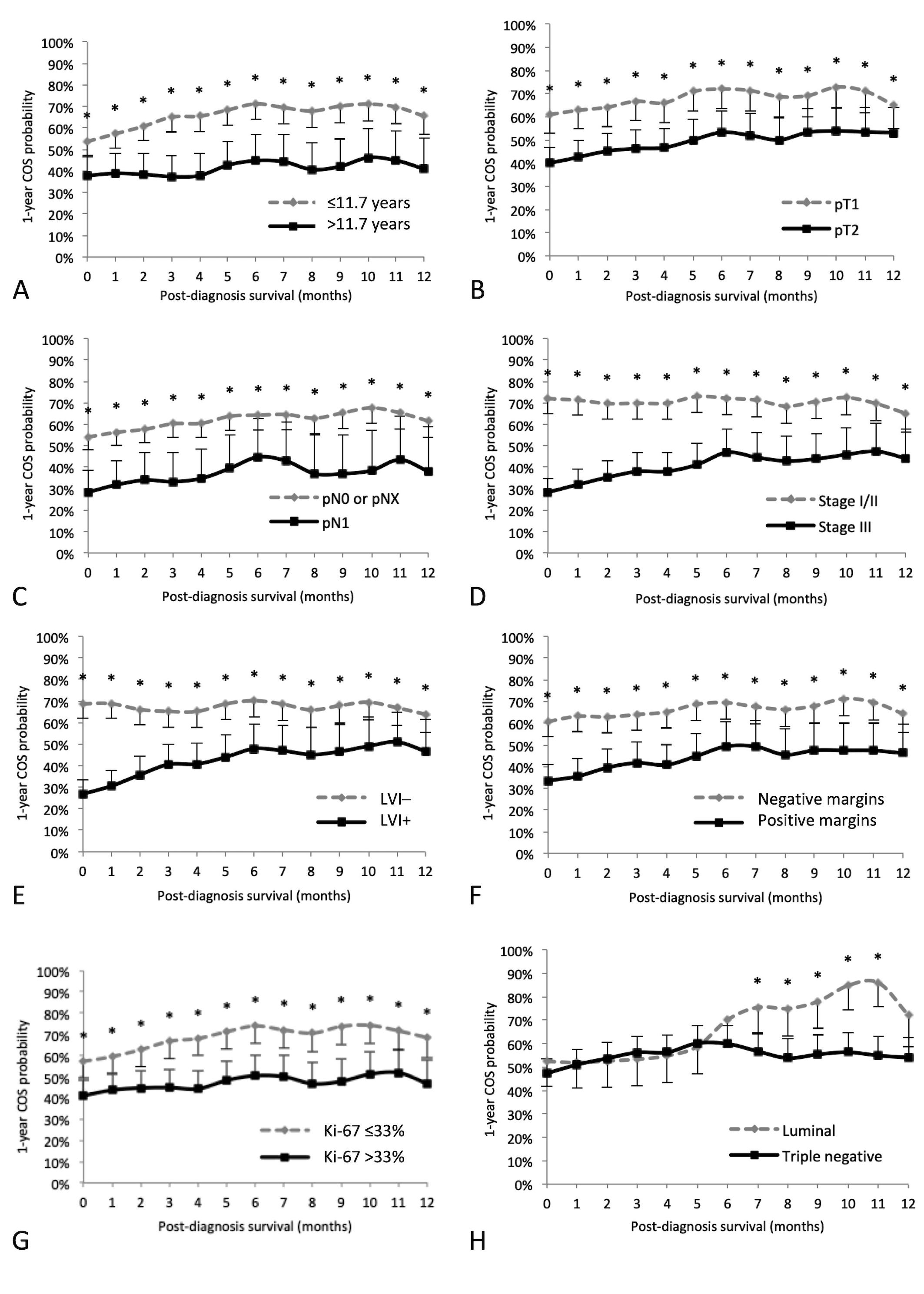
**

**Supplementary Figure 2.**


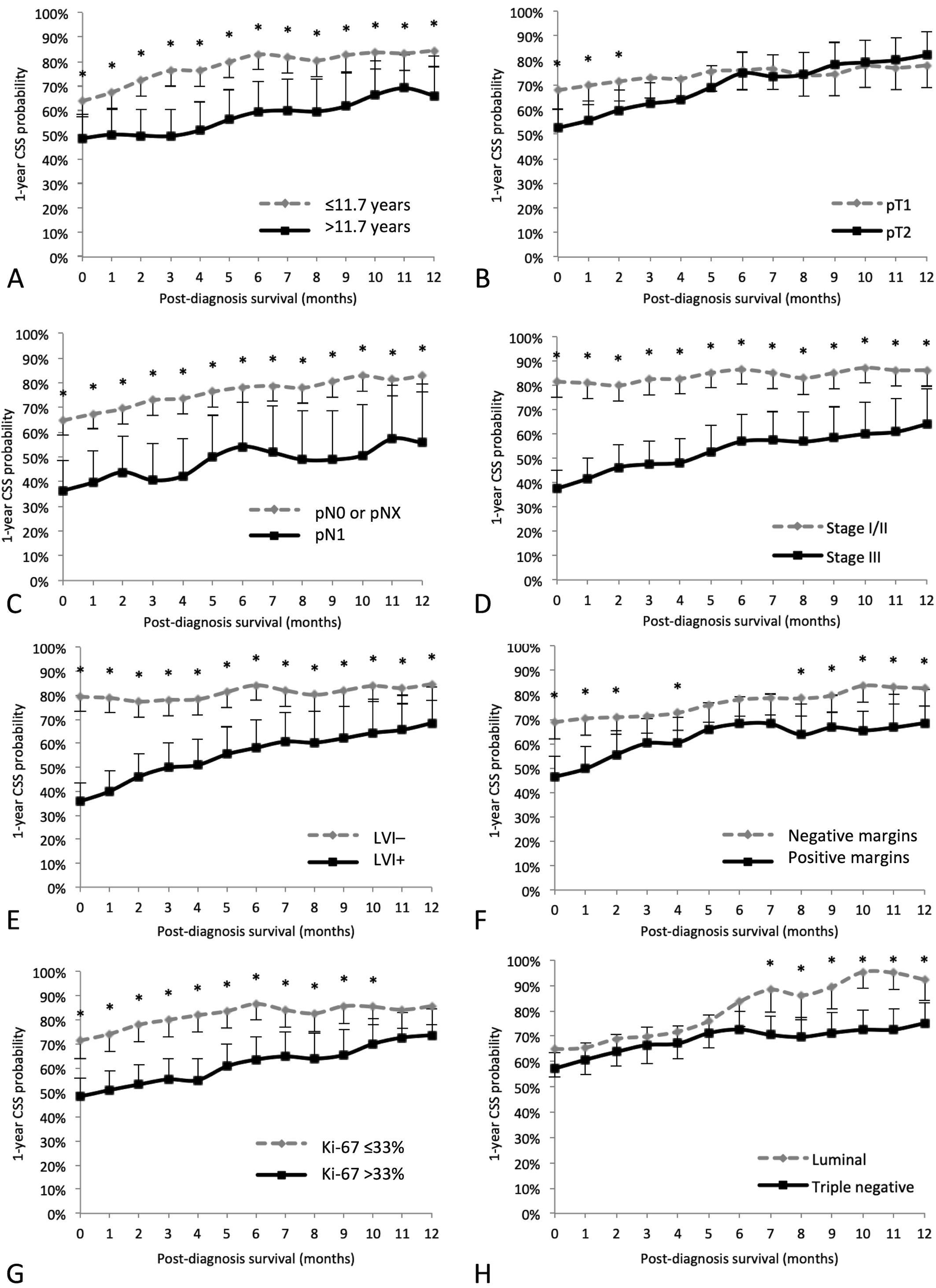


**Supplementary Figure 3.**


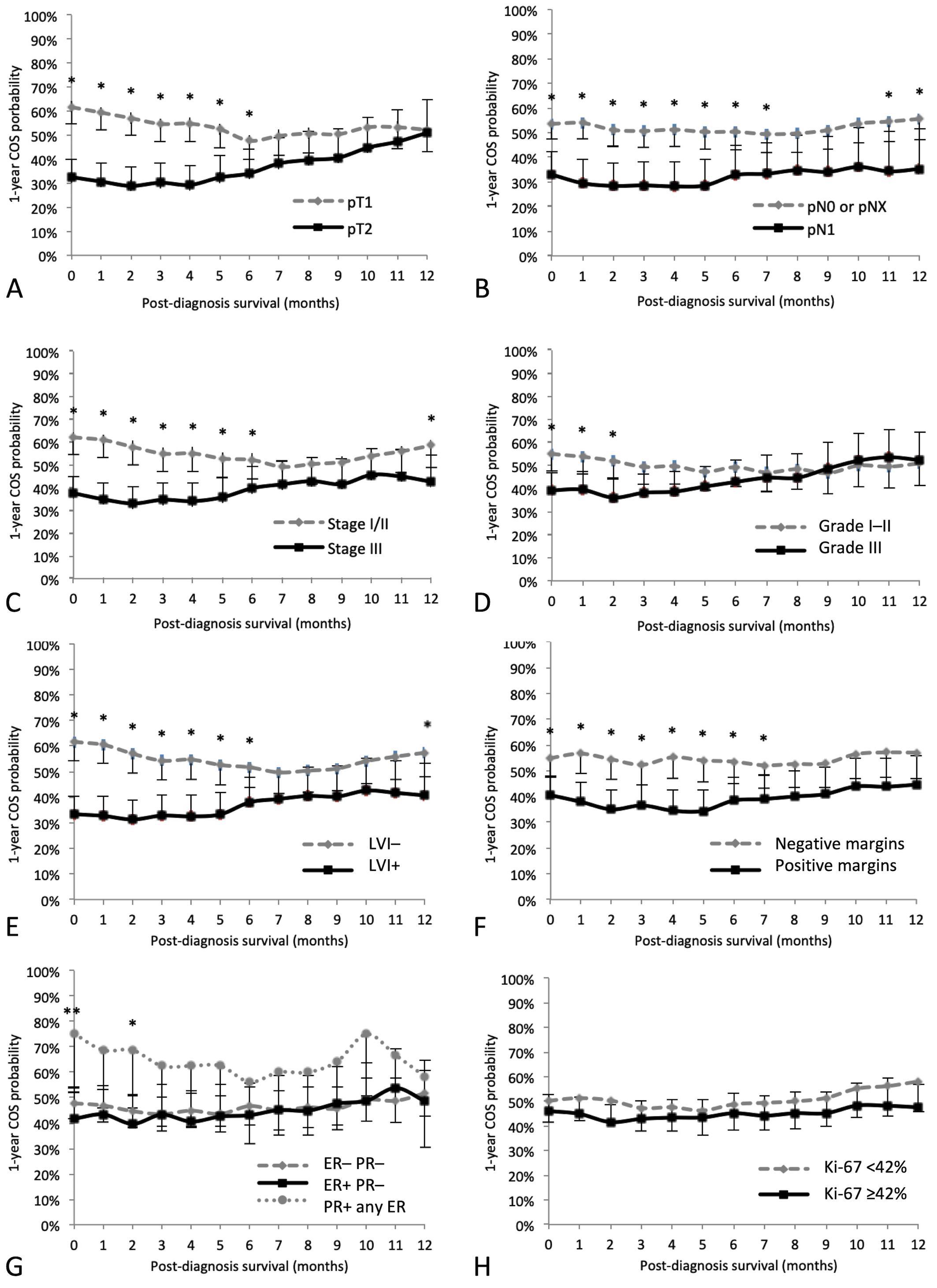


**Supplementary Figure 4.**


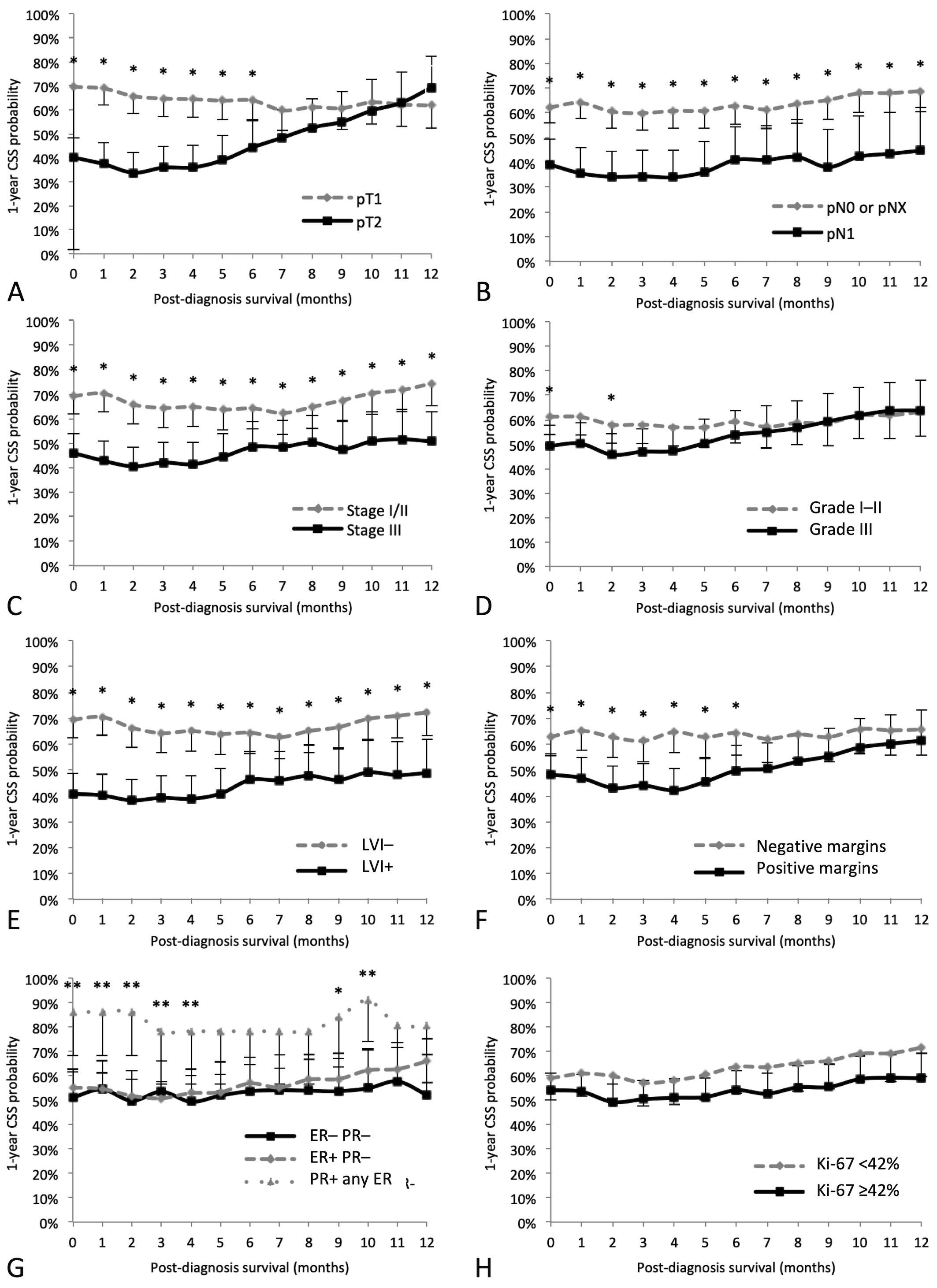

Supplement: Supplementary file 5 — Appendix S1: Supporting information [file VCO-19-140-s005.docx]
